# Benchmark Study of Redox Potential Calculations for Iron–Sulfur Clusters in Proteins

**DOI:** 10.1021/acs.inorgchem.1c03422

**Published:** 2022-04-11

**Authors:** Sonia Jafari, Yakini A. Tavares Santos, Justin Bergmann, Mehdi Irani, Ulf Ryde

**Affiliations:** † Department of Chemistry, University of Kurdistan, 66175-416 Sanandaj, Iran; ‡ Department of Theoretical Chemistry, Chemical Centre, Lund University, P.O. Box 124, SE-221 00 Lund, Sweden

## Abstract

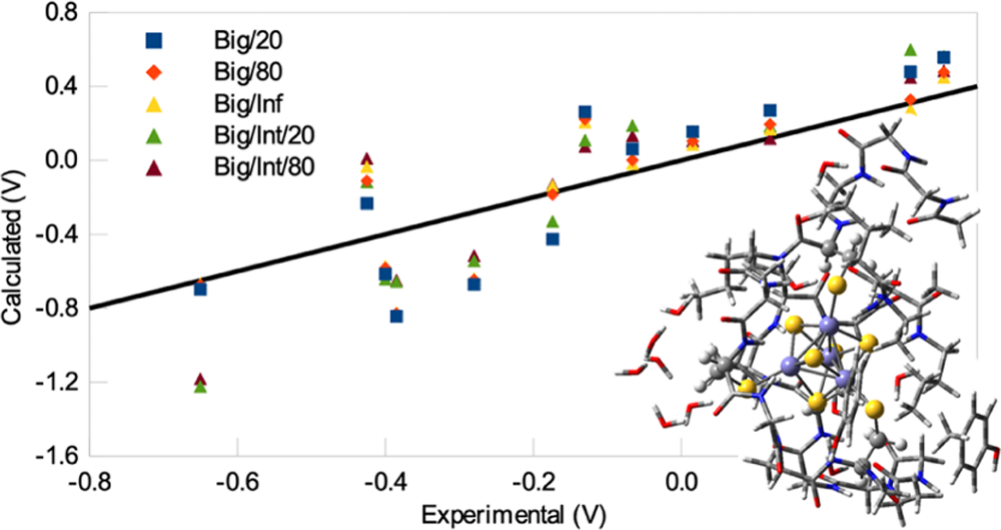

Redox potentials have been calculated for 12 different iron–sulfur sites of 6 different types with 1–4 iron ions. Structures were optimized with combined quantum mechanical and molecular mechanical (QM/MM) methods, and the redox potentials were calculated using the QM/MM energies, single-point QM methods in a continuum solvent or by QM/MM thermodynamic cycle perturbations. We show that the best results are obtained with a large QM system (∼300 atoms, but a smaller QM system, ∼150 atoms, can be used for the QM/MM geometry optimization) and a large value of the dielectric constant (80). For absolute redox potentials, the B3LYP density functional method gives better results than TPSS, and the results are improved with a larger basis set. However, for relative redox potentials, the opposite is true. The results are insensitive to the force field (charges of the surroundings) used for the QM/MM calculations or whether the protein and solvent outside the QM system are relaxed or kept fixed at the crystal structure. With the best approach for relative potentials, mean absolute and maximum deviations of 0.17 and 0.44 V, respectively, are obtained after removing a systematic error of −0.55 V. Such an approach can be used to identify the correct oxidation states involved in a certain redox reaction.

## Abstract 2

We have studied redox potentials of 12 iron−sulfur sites of 6 types with 1−4 iron ions. Structures were optimized with combined quantum mechanical and molecular mechanical (QM/MM) methods, and the redox potentials were calculated with QM/MM, QM calculations in a continuum solvent or by QM/MM thermodynamic cycle perturbations. The best results are obtained with the second approach using ∼300 atoms in the QM model and a large dielectric constant.

## Introduction

During the latest 3 decades, computational chemistry has become an important complement to experiments to study biochemical systems due to the explosive development of electronic computers and efficient software. Nowadays, relative ligand-binding affinities can be calculated with an accuracy of 4 kJ/mol for favorable cases,^1−4^ and quantum mechanical (QM) calculations have become powerful approaches to deduce and compare the reaction mechanism of enzymes.^5−7^

However, the success of calculations of accurate reduction potentials of buried groups inside proteins has been more restricted.^8−18^ In fact, even with all-atom density functional theory (DFT)/molecular dynamics (MD) simulation and free-energy perturbation (FEP) methods, the accuracy of redox potentials is not better than ∼0.26 V.^19^ Moreover, variations of methods often give rise to strongly varying results.^20,21^ The prime problem in such calculations is that redox reactions involve changing the net charge of the studied system. This gives rise to very large and long-ranged Coulombic interactions. For example, the change in the interaction energy between a site that changes its net charge by 1 *e* and a water-like oxygen atom (with a partial charge of −0.8 *e*) is 222 kJ/mol (2.3 V) at a distance of 5 Å and still 22 kJ/mol (0.23 V) at a distance of 50 Å. Fortunately, the effect is damped by the dielectric screening of the surroundings. However, with the typical dielectric constant for proteins (ε = 4), the effect is still 6 kJ/mol (48 mV) at 50 Å. This explains why the results will be extremely sensitive to the charge model used for the surrounding protein and to the accuracy of the employed protein structure. To this comes the problem that different DFT methods often give strongly diverging results for redox potentials (differences of up to 100 kJ/mol, typically depending on the amount of Hartree–Fock exchange).^14,19,22−25^

Numerous studies of redox potentials in proteins have been published.^8,9,11,26−28^ Three main approaches have been used: QM cluster calculations in a continuum solvent, continuum solvent calculations including the entire protein, employing numerical solutions to the Poisson–Boltzmann (PB) equation or other continuum or lattice-based methods, or explicitly solvated simulations with energies calculated using FEP methods. QM cluster calculations give redox potentials of small-molecule solvated metal clusters that agree with experiments within 0.2–1.6 V, although better results can occasionally be obtained, often with specific corrections.^14−18,29^ The performance of various DFT methods for the calculation of the electronic part of the redox potential has been studied for a number of simple Fe^2+/3+^ models, with mainly water ligands, by comparing to accurate CCSD(T) energies.^30^

Most previous studies have been retrospective, that is, investigating whether the computational methods can reproduce a measured redox potential or not. However, it would be more satisfying if the calculations were also predictive. Then, the calculations could be used to determine the redox state of metal centers in proteins or to design new sites with proper redox potentials. To this aim, the expected accuracy of the method needs to be known.

In biological systems, three types of metal sites are used for direct electron transfer, cytochromes, blue copper protein, and iron–sulfur clusters.^31^ The latter show the largest variation and the largest span in redox potentials (−0.7 to +0.5 V; all potentials in this article are with respect to the standard hydrogen electrode).^31,32^ The simplest FeS site is found in the rubredoxins, which contain a single Fe ion, coordinated with four cysteine (Cys) residues.^33,34^ The metal can be either in the Fe(II) or the Fe(III) oxidation states, and the redox potential varies from −0.1 to 0.1 V. The [2Fe–2S] ferredoxins contain two Fe ions connected to two bridging sulfide ions and each coordinated with two Cys residues.^35,36^ In the oxidized state, both Fe ions are in the Fe(III) state, whereas one of the Fe ions is in the Fe(II) state in the reduced state.^37^ They have redox potentials in the range of −0.45 to −0.2 V.^38^ The Rieske site is a variation of the [2Fe–2S] ferredoxins, in which one of the Fe ions is coordinated by two histidine (His) residues instead of Cys.^39^ This gives a much more positive redox potential (−0.1 to +0.3 V),^31,40^ although the site employs the same redox couples. The [4Fe–4S] ferredoxins^41−43^ contain four Fe ions bridged by four μ_3_-coordinating sulfide ions in a cubic structure. In addition, each Fe ion also coordinates to a Cys residue.^44^ They employ the Fe_3_^II^Fe_1_^III^/Fe_2_^II^Fe_2_^III^ redox couple, giving redox potentials of −0.7 to −0.3 V.^45^ The high-potential iron proteins (HiPIP) have the same cubane structure,^46,47^ but they use instead the Fe_2_^II^Fe_2_^III^/Fe_1_^II^Fe_3_^III^redox couple, giving them a much more positive potential (+0.05 to +0.5 V).^45^ Finally, the [3Fe–4S] ferredoxins have a distorted cubane structure, in which one Fe ion and one Cys residue are missing.^41,48^ They employ the Fe_1_^II^Fe_2_^III^/Fe_3_^III^ redox couple and have redox potentials in the range of −0.4 to −0.1 V.^31,45^ More complicated iron–sulfur clusters exist in some proteins (e.g., the Fe_8_S_7_Cys_6_ P-cluster), sometimes connected to catalytic functions (e.g., in the hydrogenases and nitrogenases).^49,50^

The redox potentials of iron–sulfur clusters have been studied with many computational methods previously. Early studies used pure QM^25,51−55^ or pure electrostatics.^56,57^ Several studies used QM calculations with continuum solvation.^51,58−61^ Noodleman and co-workers have calculated redox potentials for many iron–sulfur clusters of different types using QM calculations of the active sites (including hydrogen bonds to the metal ligands) to get absolute potentials as well as charges.^11,26,27,62,63^ The latter are then used in a PB calculation of solvation energies, including the entire proteins. They report errors of 0.2–0.6 V for the absolute potentials but 0.07–0.11 V in relative potentials. However, for the FeMo cluster of nitrogenase, this approach gave a redox potential that was 1.3 V too negative, leading to an incorrect prediction of the nature of the (at that time unknown) central atom.^64^ Similar PB-based calculations have also been used by other groups.^65−69^ In particular, Ichiye and co-workers have reported mean errors of only 0.03–0.07 V for iron–sulfur sites of the same type.^70−72^

In this investigation, we calculate redox potentials of 12 different iron–sulfur clusters with 1–4 Fe ions using QM cluster calculations in a continuum solvent^73^ based on QM/molecular mechanical (MM) structures.^5,20,74−80^ We test several variations of the approach in terms of the QM method, the basis set, the size of the QM system, and the details in the QM/MM calculations. We also include QTCP (QM/MM thermodynamic cycle perturbation) calculations^81−83^ to study the effect of including dynamics. The study aims to deduce which of the tested methods is most accurate and if they are accurate enough to be used for predictive studies.

## Methods

### Studied Systems

We have studied 12 iron–sulfur clusters of 6 different types in 11 crystal structures.^33−36,39,41−43,46−48^ The systems, the experimental redox potentials, and the employed crystal structures are described in Table 1. Two rubredoxins with mononuclear Fe(Cys)_4_ clusters were studied, from *Clostridium pasteurianum* (1IRO; Rub1)^33^ and *Pyrococcus furiosus* (5NW3; Rub2),^34^ with redox potentials that differ by ∼80 mV. Two [2Fe–2S] ferredoxins were studied from *Nostoc* sp. PCC 7119 (1QT9; 2Fd1)^36^ and *Burkholderia cepacia* (2PIA; 2Fd2),^35^ with a difference in the redox potentials of 231 mV. A Rieske site (Cys_2_FeS_2_FeHis_2_) from *Rhodobacter sphaeroides* (2NUK; Rieske) was studied.^39^ The experimental redox potential of the Rieske site is pH dependent, and we use the potential obtained at neutral pH, indicating that both His ligands are neutral in both the reduced and oxidized states.^63^ [3Fe–4S] ferredoxins were studied from *Desulfovibrio gigas* (1FXD; 3Fd1)^48^ and *Azotobacter vinelandii* (5FD1; 3Fd2)^41^ with differences in the potential of 295 mV. [4Fe–4S] ferredoxins were studied from *Bacillus thermoproteolyticus* (1IQZ; 4Fd1),^42^*Desulfovibrio africanus* (1FXR; 4Fd2),^43^ and *A. vinelandii* (5FD1; 4Fd3)^41^ with differences in the potential of 370 mV. Finally, HiPIP sites were studied from *Allochromatium vinosum* (1CKU; Hip1)^47^ and *Halorhodospira halophila* (2HIP; Hip2),^46^ with a difference in the potential of 235 mV. In total, the considered redox potentials cover a range of 1005 mV from −650 to 355 mV.

**Table 1:** Studied Systems, Describing the FeS Site, the Source, the Abbreviation Used in the Article (abb), the Crystal Structures (Protein Databank Code; PDB) Used for the Simulations and Their Resolution (res) in Å, the Experimental Reduction Potential (*E*°), the Number of Fe(II) Ions (Formally) in the Reduced State (*n*_red_^II^), as Well as the Spin State for the Reduced and Oxidized States (*S*_red_ and *S*_ox_)

site	organism	abb	PDB	res	*E*° (mV)	*n*_red_^II^	*S*_red_	*S*_ox_
rubredoxin	*C. pasteurianum*	Rub1	1IRO(33)	1.1	–66^a^^31^	1	2	5/2
	*P. furiosus*	Rub2	5NW3^34^	0.59	16^a^^31^	1	2	5/2
[2Fe–2S] ferredoxin	*Nostoc* sp. PCC 7119	2Fd1	1QT9^36^	1.3	–405^90^	1	1/2	0
	*B. cepacia*	2Fd2	2PIA(35)	2	–174^35^	1	1/2	0
Rieske	*R. sphaeroides*	Rieske	2NUK(39)	1.2	310^68^	1	1/2	0
[3Fe–4S] ferredoxin	*Desulfovibrio gigas*	3Fd1	1FXD(48)	1.7	–130^91^	1	2	1/2
	*A. vinelandii*	3Fd2	5FD1(41)	1.9	–425^92^	1	2	1/2
[4Fe–4S] ferredoxin	*B. thermoproteolyticus*	4Fd1	1IQZ(42)	0.92	–280^93^	3	1/2	0
	*D. africanus*	4Fd2	1FXR(43)	2.3	–385^94^	3	1/2	0
	*A. vinelandii*	4Fd3	5FD1(41)	1.9	–650^92^	3	1/2	0
HiPIP	*A. vinosum*	Hip1	1CKU(47)	1.2	355^a^^95,96^	2	0	1/2
	*H. halophila*	Hip2	2HIP(46)	2.5	120^97^	2	0	1/2

a Average of two experimental values.

Each protein was set up starting from the crystal structure specified in Table 1. For each structure, all heteromolecules were removed, except the Fe–S clusters. However, for the 2PIA structure, we kept the flavin mononucleotide. We used only the first chain in the calculations for the dimeric protein structures (1FXR, 1CKU, and 2HIP). For residues with alternative conformations, we kept the one with the highest occupation number or the first if they have equal occupation numbers. All crystal water molecules were kept. The protonation states of all titratable protein residues were determined by a detailed study of the hydrogen bond pattern, the solvent accessibility, and the possible formation of ionic pairs. They were also checked by PROPKA calculations^84−86^ and by the suggestions of Maestro software.^87^ All Asp, Glu, Arg, and Lys residues were assumed to be charged unless they are buried inside the proteins and the hydrogen bond pattern suggests that they are neutral. Hence, Glu-223 in the 2PIA structure was neutralized, whereas all other Asp, Glu, Arg, and Lys residues in all proteins were assumed to be charged. A thorough manual investigation of all His residues gave the protonation assignment detailed in Table S1 in the Supporting Information. All Cys residues were protonated, except those coordinated to the Fe ions or making a disulfide bridge with each other (cf. Table S1). A modified Cys residue in the 1FXD structure (Cys-11), which was tentatively interpreted as containing an extra −SCH_3_ group,^48^ was changed back to a normal Cys residue, in agreement with NMR studies.^88^ The Maestro software^87^ was used to suggest Asn and Gln residues in which the side-chain N and O atoms are flipped, and it was also used to add all protons to the crystal structures. However, one of the systems was taken from a previous QM/MM study (1CKU).^89^

After protonation, the proteins were immersed in a periodic truncated octahedral box of TIP3P water molecules,^98^ extending at least 10 Å from the solute using the *tleap* program in the Amber software suite.^99^ Next, the hydrogen atoms and the added water molecules were subjected to 1000 cycles of minimization with the heavy atoms of the proteins restrained. This was followed by a 10 ps constant volume equilibration with the same restraints. Finally, the systems were equilibrated by a 1 ns constant volume simulation and a 1 ns simulated annealing at constant pressure with the same restraints (the force constant for the restraints in all steps was 1000 kcal/mol/Å^2^). Bond lengths involving hydrogen atoms were constrained by the SHAKE algorithm^100^ (not in the minimizations), allowing for a time step of 2 fs during the simulations. The temperature was kept constant at 300 K using Langevin dynamics with a collision frequency of 2 ps^–1^.^101^ The pressure was kept constant at 1 atm using Berendsen’s weak coupling isotropic algorithm with a relaxation time of 1 ps.^102^ Long-range electrostatics were handled by particle mesh Ewald summation^103^ with a fourth-order B-spline interpolation and a tolerance of 10^–5^. The cut-off radius for Lennard-Jones interactions was set to 8 Å. After the final equilibration, the octahedral systems were truncated to a spherical shape with the largest radius that fits into the spherical system around the geometric center of the proteins.

### QM Calculations

QM calculations were performed using Turbomole software.^104^ Two DFT methods (TPSS and B3LYP)^105−108^ and three different basis sets were used (def2-SV(P), def2-TZVPD, and aug-cc-pVTZ).^109^ We selected one meta generalized gradient approximation functional and one hybrid functional with 20% Hartree–Fock exchange to judge the importance of the exact exchange on the calculated redox potentials. Hybrid functionals normally give better results than pure functionals for most systems, but the opposite is sometimes found for redox potentials of metal complexes.^15^ The calculations were sped up by expanding the Coulomb interactions in an auxiliary basis set, the resolution-of-identity approximation.^110,111^ Empirical dispersion corrections were included with the DFT-D3 approach^112^ and Becke–Johnson damping,^113^ as implemented in Turbomole.

In some calculations, the QM system was immersed into a continuum solvent, employing the conductor-like screening model (COSMO),^114,115^ implemented in Turbomole. The default optimized COSMO atomic radii and a water solvent radius of 1.3 Å were employed to construct the solvent-accessible surface cavity,^116^ whereas radii of 2.0 and 2.11 Å were used for Fe and P, respectively.^117^ Structures for the QM + COSMO calculations were taken directly from the QM/MM calculations (next section) without further optimization. The dielectric constant of proteins has been much discussed, but typically values of 4–20 are used.^9,12^ We have tested three values, 4, 20, and 80 (in one case, also an infinite dielectric constant).

**Figure 1:**
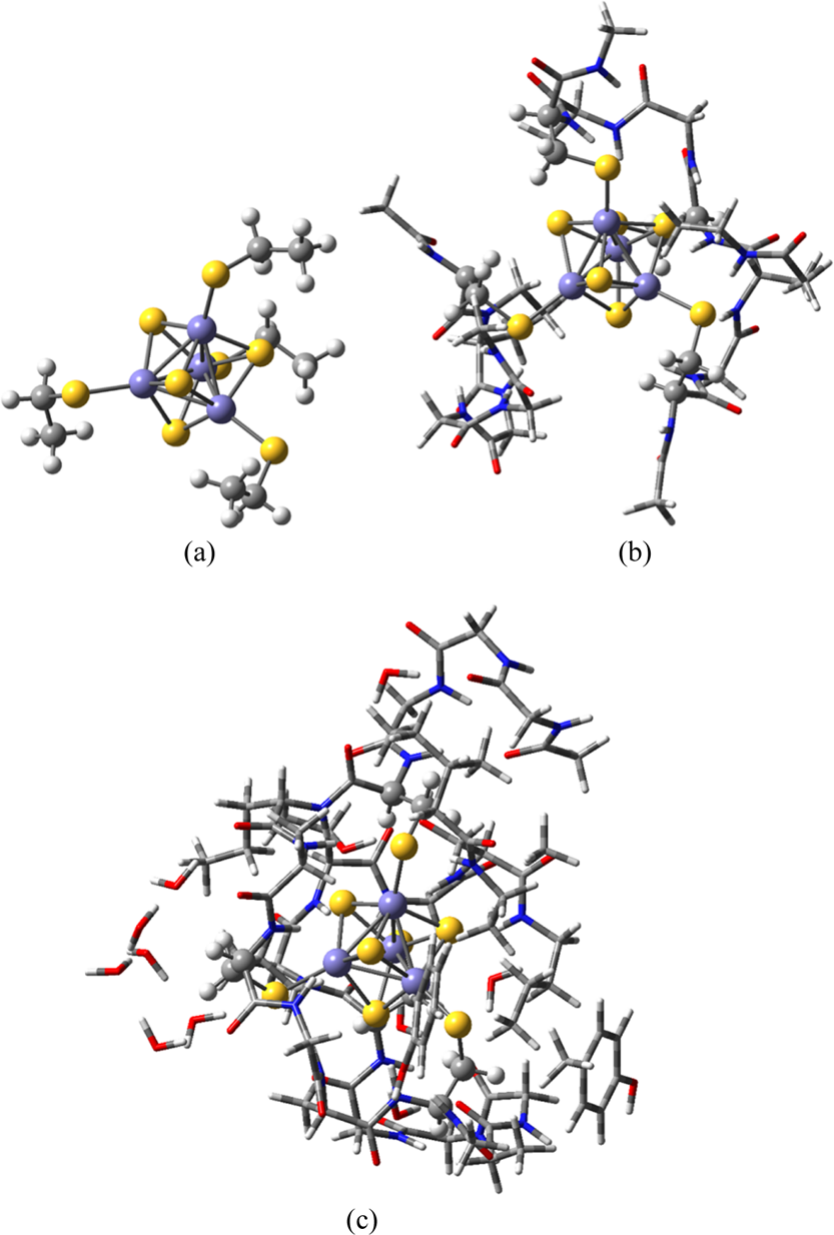
QM systems for 4Fd1: (a) Min, (b) Int, and (c) Big. The minimal system is shown by balls in the center of the Int and Big systems.

Three different sizes of the QM systems were employed. The minimal QM system (Min) consisted of the Fe and S ions, as well as the directly coordinated Cys or His groups, modeled by CH_3_CH_2_S^–^ and methylimidazole, respectively. In the intermediate QM system (Int), all groups forming hydrogen bonds to the Cys or S^2–^ ligands were also included. Backbone amide groups were modeled by CH_3_CONHCH_3_, whereas protein side chains were modeled by the corresponding functional groups, truncated by a methyl group. The Cys and His ligands were extended to include the whole residue, including the CH_3_CO– and −NHCH_3_ groups from the preceding and following residues, respectively. The largest QM system (Big) included all functional groups in the proteins with any atom within 3.5 Å of the minimal QM system. These QM systems were set up using our local program for big-QM calculations (changepdb).^118^ The sizes of the three types of the QM systems for all proteins are given in Table S1 in the Supporting Information. As an example, the Min, Int, and Big QM systems of the 4Fd1 system consisted of 40, 156, and 306 atoms, respectively (including hydrogen link atoms), and they are shown in Figure 1.

Redox potentials (*E*°) were calculated according to
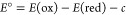
1where *E*(ox) and *E*(red) are the energies of the oxidized and reduced states and *c* is a correction factor (4.28 eV) to place the potentials on the scale of the standard hydrogen electrode.^119^ The actual value of this factor has been much discussed, and values between 4.05 and 4.44 eV have been suggested,^119,120^ but it has little influence on our results because only one of our seven quality criteria (see below) is affected by this factor.

For the rubredoxin systems, we studied the redox potentials between the Fe^II^ and Fe^III^ states. For the [2Fe–2S] ferredoxin and Rieske sites, we studied the potential between the Fe_1_^II^Fe_1_^III^ and Fe_2_^III^ redox states. For [4Fe–4S] ferredoxin, we studied the transition between the Fe_3_^II^Fe_1_^III^ and Fe_2_^II^Fe_2_^III^ redox states, whereas for HiPIP, it was instead the Fe_2_^II^Fe_2_^III^ and Fe_1_^II^Fe_3_^IIII^ states. Finally, for [3Fe–4S] ferredoxin, we considered the transition between the Fe_1_^II^Fe_2_^IIII^ and Fe_3_^IIII^ redox states.

The electronic structures of the iron–sulfur clusters are complicated. Each Fe ion is in the high-spin state (five and four unpaired electrons for Fe(III) and Fe(II), respectively). However, these spins are coupled antiferromagnetically to a lower spin state in the polynuclear clusters, typically *S* = 0 or 1/2,^121,122^ (but *S* = 2 for the reduced [3Fe–4S] clusters^122^), as is specified in Table 1. Such antiferromagnetically coupled sites are normally described by the broken-symmetry (BS) approach in DFT calculations.^11,123^ For the [2Fe–2S] clusters (including the Rieske sites), there is only a single possible BS state. However, there are six possible BS states for the [4Fe–4S] clusters (two sites with dominant beta spin can be selected out of four sites in six different ways). For the [3Fe–4S] clusters, there are three possible BS states. We examined all possibilities and selected that with the most favorable energy for the minimal QM system of each protein and oxidation state. This BS state was also used for the other calculations.

The BS states were either generated by the fragment approach of Szilagyi and Winslow^124^ or by obtaining one BS state by first optimizing the highest possible spin state (all unpaired electrons aligned), flipping the spins to the desired state, and then obtaining the other BS states by simply swapping the coordinates of the Fe ions.^125^ No attempt was made to use spin projection corrections to the BS energies^26^ because such schemes are often problematic for non-symmetric protein systems,^89^ and the effect typically cancels for the calculation of redox potentials.^52−54^ For all methods, we have ignored the zero-point energy, enthalpic, or entropy corrections because a frequency calculation becomes prohibitively expensive for the large models, and such effects have been shown to be small in electron-transfer reactions.^126−128^

### QM/MM Calculations

The QM/MM calculations were performed with ComQum software.^129,130^ In this approach, the protein and solvent are split into three subsystems: system 1 (the QM region) was relaxed by QM methods, whereas system 2 contained all atoms in residues and water molecules with at least one atom within 6 Å of any atom in the QM system and was optionally optimized by MM in each step of the QM/MM geometry optimization. System 3 involved the remaining part of the protein and the solvent and was kept fixed at the original coordinates (equilibrated crystal structure).

In the QM calculations, system 1 was represented by a wave function, whereas all the other atoms were represented by an array of partial point charges, one for each atom, taken from the MM setup. Thereby, the polarization of the QM system by the surroundings is included in a self-consistent manner. When there is a bond between systems 1 and 2, the hydrogen link atom approach was employed: the QM system was capped with hydrogen atoms (hydrogen link atoms, HL), whose positions are linearly related to the corresponding carbon atoms (carbon link atoms, CL) in the full system.^129,131^ All atoms were included in the point charge model, except the CL atoms.^132^

The total QM/MM energy in ComQum was calculated as^129,130^
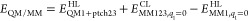
2where *E*_QM1+ptch23_^HL^ is the QM energy of system 1 truncated by HL atoms and embedded in a set of point charge modeling of systems 2 and 3 (but excluding the self-energy of the point charges).  is the MM energy of system 1, still truncated by HL atoms, but without any electrostatic interactions. Finally,  is the classical energy of all atoms in the model with CL atoms and with the charges of the QM region set to zero (to avoid double-counting of the electrostatic interactions). Thus, ComQum employs a subtractive scheme with electrostatic embedding and van der Waals link atom corrections.^133^ No cut-off is used for any of the interactions in the three energy terms in eq 2.

The geometry optimizations were continued until the energy change between two iterations was less than 2.6 J/mol (10^–6^ a.u.) and the maximum norm of the Cartesian gradients was below 10^–3^ a.u.

### QTCP Calculations

The QTCP approach is a method to calculate free energies between two states, Red and Ox, with a high-level QM/MM method, using FEP and sampling at only the MM level.^81−83^ It employs a thermodynamic cycle, showing that the QM/MM free-energy difference between Red to Ox states can be obtained from three calculations: an FEP from Red to Ox at the MM level and two FEP calculations in the method space from MM to QM/MM, one each for the Red and Ox states
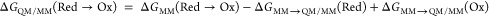
3

The QTCP calculations were performed as has been described before.^83,134^ First, each oxidation state was optimized by QM/MM, keeping systems 2 and 3 fixed at the equilibrated crystal structure. Then, the protein was further solvated in an octahedral box of TIP3P water molecules,^98^ extending at least 9 Å from the QM/MM system. For the Red state, the whole system was subjected to a 1000-step minimization, keeping the atoms in the QM region fixed and restraining all heavy protein atoms with a force constant of 100 kcal/mol/Å^2^. Then, two 20 ps MD simulations were run with the heavy protein atoms still restrained. The first was run with a constant volume and the second with a constant pressure. Next, a constant pressure MD simulation equilibrated the size of the periodic box for 100 ps, and only the heavy atoms in the QM system were restrained to the QM/MM structure. The final structure of this simulation was used as the starting structure also for the Ox state. Finally, an equilibration of 200 ps and a production simulation of 400 ps were run with a constant volume for both Red and Ox states. During the production run, 200 snapshots were collected every 2 ps. Based on these snapshots, three sets of FEPs were performed. First, charges were modified in nine steps from those of the Red state to those of the Ox state, keeping the coordinates to those of the Red state. Second, the coordinates were modified in five steps to those of the Ox state, with the charges of the Ox state. Finally, MM → QM/MM FEPs were performed for both the Red and Ox states, keeping the QM systems fixed, as has been described before.^82,83^ All FEP calculations were performed with the local software, *calcqtcp*.

The MD simulations were performed for periodic systems in the geometry of a truncated octahedron. We tested three options for considering the long-range electrostatic effects in the FEP calculations. In the first, we ignored the periodicity and calculated only interactions within the primary box with no cut-off. Long-range solvation effects were calculated by the Born/Onsager equation. This is called Born calculations in the following. In the second approach, we used the periodicity and calculated all electrostatic interactions with Ewald summation and Lennard-Jones interactions with a cut-off of 8 Å. This approach is called Ewald. In the third approach, electrostatic interactions were instead calculated with the generalized Born approach by Onufriev, Bashford, and Case (*igb* = 5 in Amber)^135^ for the non-periodic system. This will be called GB in the following.

Previous studies have shown that FEP calculations in an explicit solvent still overestimate the effect from solvent-exposed charged groups.^134,136^ QTCP has the option to ignore these groups in the FEP calculations. This was also tested. Moreover, in one series of QTCP calculations, we neutralized charged residues (Glu, Asp, Arg, and Lys) on the surface of the studied proteins.^20,136^ This was performed by protonating the Glu and Asp residues on the OE2 and OD2 atoms, respectively. For the Arg and Lys residues, we removed a proton from their NH2 and NZ atoms, respectively. Then, we equilibrated the hydrogen atoms and added water molecules in the systems, assigned the most stable BS state, optimized the minimal QM system using QM/MM calculations, and performed the QTCP calculations as described above.

### Quality Measures

Each method was evaluated by several quality measures. First, we calculated the correlation coefficient (*R*^2^), the mean absolute deviation (MAD), the MAD after removing systematic error (i.e., the mean signed error, MSE; MADtr), and maximum error (also after removal of the systematic errors; MAXtr), all compared to the experimental redox potentials in Table 1. Moreover, we calculated Spearman’s rank correlation coefficient (ρ) and Kendall’s τ to investigate how well the various methods get the correct order of the redox potentials. The latter was calculated both for the seven pairs of potentials for iron–sulfur sites of the same type (one pair each for rubredoxin, [2Fe–2S], [3Fe–4S], and HiPIP, and three for [4Fe–4S]; called τ_7_) and for all the 66 pairs of redox potentials (the experimental potentials show a reasonably uniform distribution with differences larger than 0.04 V for all except two pairs, so we decided not to exclude any pair for this measure;^137^ called τ_66_). Finally, we considered the range of the 12 calculated redox potentials compared with the experimental range, which is 1.005 V.

To get a single score to compare the methods, we calculated the rank of each method among all the methods for the range, MAD, MADtr, MAXtr, *R*^2^, ρ, and τ_66_ and summed these seven ranks. This sum was then used to rank all the methods.

It should be noted that the quality measures fall into two groups with often opposing trends. *R*^2^, ρ, and τ_66_ indicate how well the various methods can rank the different sites. *R*^2^ is sensitive to the largest and smallest potentials, and all the three are often good for methods that overestimate the magnitude of the redox potentials. On the other hand, the range, MAD, MADtr, and MAXtr measure the absolute values of the redox potentials and penalize methods that give too large magnitudes and differences between the various redox sites. They may give good results for methods that underestimate the potentials. For example, setting all potentials to 0 V gives a MAD of 0.3 V, a MADtr of 0.24 V, and a MAXtr of 0.5 V, a quite good result (but the range, *R*^2^, ρ, and τ_66_ all vanish). Therefore, it is important to employ quality measures of both types in the final score.

## Results and Discussion

In this investigation, we have tried to reproduce experimental redox potentials for a set of 12 different iron–sulfur clusters with computational methods. The test set was selected to cover the most common types of FeS clusters, viz. rubredoxin , [2Fe–2S] ferredoxins, Rieske clusters, [3Fe–4S] ferredoxins, [4Fe–4S] ferredoxins, and HiPIPs. The test cases were selected based on the availability of good crystal structures and measured redox potentials. Moreover, we tried to obtain two proteins for each type with as different experimental redox potentials as possible. The test cases are described in Table 1.

The redox potentials were obtained by three general methods: QM/MM,^5,20,74−80^ QM cluster^73^ calculations in a COSMO continuum solvent,^114,115^ and QTCP^81−83^ (but all structures were obtained by QM/MM and the TPSS/def2-SV(P) basis set). For each general method, we tested several variants:

- Two different DFT methods, TPSS-D3 or B3LYP-D3, combined with the def2-SV(P), def2-TZVPD, or aug-cc-pVTZ basis sets.
- Three sizes of the QM system: Min, Int, and Big (cf. Figure 1).
- Two different force fields were used in the QM/MM optimizations (involving different atomic charges of the protein residues): FF14SB^138^ or FF15IPQ.^139^
- Two different QM/MM optimization approaches, regarding the relaxation of the surrounding protein and solvent (system 2): fixed or relaxed.
- Three different dielectric constants for the COSMO calculations: ε = 4, 20, or 80.
- Three different approaches to calculate long-range electrostatic effects in the QTCP calculations (Born, Ewald, or GB) and two different treatments of the solvent-exposed charges (included or excluded). We also run one set of calculations in which the solvent-exposed charged groups were neutralized already in the QM/MM optimizations and MD simulations.

These variations of the methods gave us 113 data sets (25 QM/MM, 24 QTCP and 64 QM + COSMO), each set with 12 redox potentials (1356 calculated redox potentials in total). Each method was evaluated by seven quality measures, the range, MAD, MADtr, MAXtr, *R*^2^, ρ, and τ_66_. The sum of the ranks for each method among the considered methods for these seven measures was used as our final quality measure.

The calculated redox potentials for all methods are presented in Tables S2–S5 in the Supporting Information. Quality measures of the tested methods are listed in Tables 2–4. The results of the four types of methods will be discussed in separate sections in the following.

### Geometries

Redox potentials were based on geometries obtained by QM/MM calculations. Therefore, we start by discussing these. Geometries were obtained by eight different variants of QM/MM depending on the size of the QM system (Min, Int, or Big), the force field (FF14SB or FF15IPQ), and whether the surroundings were relaxed or kept fixed at the starting crystal structure (free or fix).

The Fe–Fe and Fe–S/N distances of all systems in the optimized structures are summarized in Tables S6–S17 in the Supporting Information. The distances do not vary so much between the different variants of the QM/MM methods (the range over the eight methods are 0.05 and 0.13 Å, averaged over all proteins and all the Fe–S and Fe–Fe distances, respectively).

It is hard to decide which method reproduces the experimental structures best because the oxidation state of the Fe–S cluster in the crystal structures is not always specified and it may change during data collection (photoreduction). Moreover, several of the crystal structures are at a rather poor resolution (up to 2.5 Å). In fact, there is a good correlation between the MAD between the calculated and experimental Fe–S distances and the resolution of the crystal structure (*R* = 0.82), indicating that such a comparison evaluates the quality of the crystal structure rather than the QM/MM structure.

However, the 5NW3 structure of oxidized rubredoxin is at atomic resolution (0.59 Å).^34^ The two QM/MM structures with relaxed surroundings, Min or Int QM system, and the FF14SB force field reproduce the four Fe–S distances with a MAD of 0.008 Å. The corresponding structures with fixed surroundings are slightly worse (0.010 Å), followed by the two structures obtained with the FF15IPQ force field (0.010–0.013 Å), whereas the two calculations with the big QM system give MADs of 0.017–0.020 Å (relaxed surroundings always better than fixed).

The 1IQZ structure of 4Fd1 is also at atomic resolution (0.92 Å).^42^ The structures with the Int QM system, fixed surroundings and FF14SB, and the big QM system, relaxed surroundings, and FF14SB give the lowest MAD for the Fe–S distances (0.024 Å), but the other structures are not much worse, with MADs up to 0.035 Å and fixed surroundings are not always worse. The MAD is larger and more varying for the Fe–Fe distances. The structures that give the best Fe–S distances give the worst Fe–Fe distances, with a MAD of ∼0.10 Å.

For the Hip1 structure (1CKU, reduced) at 1.2 Å, the Min QM system with fixed surroundings gives the lowest MAD for the Fe–S distances (0.018 Å) and the Big QM system with fixed surroundings the highest MAD (0.046 Å). However, for the more flexible Fe–Fe distances, Int/FF15IPQ gives the lowest MAD 0.063 Å, whereas the Int/FF14SB calculations give MADs of 0.11 Å.

The Rieske structure (2NUK) at the same resolution gives somewhat different results. For this protein, the two Int calculations with relaxed surroundings and also the Big calculation with fixed surroundings give the lowest MAD for the Fe–S/N distances (0.03 Å), whereas Min/Fix gives the worst results (0.056 Å). It is notable that the corresponding reduced models agree better with the crystal structure for all calculations (MAD = 0.02–0.04 Å). This indicates that the crystal structure is actually photoreduced during data collection.

Finally, we compared the geometries of the reduced and oxidized states for the various proteins. The addition of one electron has a rather small influence on the structures. The Fe–S distances are ∼0.03 Å longer in the reduced state than in the oxidized state. The effect is larger for the [3Fe–4S] sites (0.06 Å) and slightly smaller for the [4Fe–4S] sites (both 4Fd and Hip), ∼0.02 Å. The variation among the eight methods is small, less than 0.03 Å.

The Fe–Fe distances typically show only small differences between the two oxidation states, less than 0.03 Å, and it can both increase or decrease upon reduction, although the latter is more common. The difference is somewhat larger for the Rieske site (around −0.05 Å).

### Spin Populations

Mulliken spin populations of the various optimized QM/MM structures are described in Table S18. They vary slightly between the different sizes of the QM system, the MM force field, or whether the surroundings are relaxed or not (typically by 0.1–0.2 e, but occasionally more for the reduced [3Fe–4S] and HiPIP sites).

For rubredoxin, the Fe spin is 3.9 e in the oxidized state and 3.6 e in the reduced state in the TPSS/def2-SV(P) calculations, with very little variations. This is appreciably lower than the formal number of unpaired spins, 5 and 4, respectively, but similar to what is found in similar DFT studies of iron–sulfur clusters.^62,89,140^ The remaining spin is distributed onto the surrounding sulfur atoms.

For the 2Fd clusters and the Rieske sites, both Fe ions have a spin population of 3.5–3.7 e (in absolute terms) in the oxidized state, but that of one of the Fe ions drops to 3.2–3.4 e in the reduced state, indicating a spin-localized state. The two Fe ions are antiferromagnetically coupled.

For 3Fd, all Fe ions have spin populations of 3.4–3.6 e (absolute) in the reduced state (formally with one Fe(II) and two Fe(III) ions), indicating that the extra electron is delocalized over the entire cluster. One Fe ion has a minority spin. However, in the oxidized state (with *S* = 1/2), the situation is different. Two Fe ions have similar but opposite spin populations of 2.3–3.5 e, whereas the third Fe ion has a much lower spin of 1.5–2.2 e.

For the 4Fd and HiPIP sites, there is little variation in the spin populations, being 3.2–3.5, 2.9–3.6, 2.6–3.6, and 3.2–3.5 e (in absolute terms) for oxidized 4Fd, reduced 4Fd, oxidized HiPIP, and reduced HiPIP, respectively. However, for reduced 4Fd and oxidized HiPIP, there is a tendency that one or two Fe ions have a lower spin population (2.6–3.3 e) than the others, but the variation is quite large between the various proteins and calculations. The two calculations with oxidized Hip1 and the big QM system are special by having low spin populations for all four Fe ions (two with 2.6–2.8 e and two with 3.1 e).

Changing the method to B3LYP in general increases the magnitude of the Fe spin populations by 0.3 e on average (least for rubredoxin and [2Fe–2S] clusters and most for [3Fe–4S] clusters). Enlarging the basis set from def2-SV(P) to def2-TZVPD has small and varying effects on the spin populations for both TPSS and B3LYP functionals (averages of −0.02 and −0.04 e for the two methods, respectively).

### QM/MM Redox Potentials

Next, we discuss the calculated redox potentials, starting with the results obtained by QM/MM. We tested 25 different variants of the QM/MM approach (different DFT methods, basis sets, sizes of the QM system, MM force fields, and whether the surroundings were allowed to relax or not) to calculate the redox potentials. However, only the eight variants with TPSS/def2-SV(P) employ geometries optimized with the same method, whereas the other variants employ single-point QM/MM calculations on structures optimized with TPSS/def2-SV(P). The calculated redox potentials from the QM/MM calculations are shown in Table S2 in the Supporting Information, and the quality measures are shown in Table 2.

**Table 2:** Quality Measures (Range, MAD, MADtr, and MAXtr in *V*; *R*^2^, ρ, τ_7_, and τ_66_) for the QM/MM Calculations^a^

method	QMS	FF	Surr	Range	MAD	MADtr	MAXtr	*R*^2^	ρ	τ_7_	τ_66_	rank
TPSS/SV opt	Min	14	fix	17.9	12.3	4.6	9.2	0.80	0.87	0.71	0.67	90
			relax	16.1	11.5	4.3	8.6	0.81	0.89	0.71	0.70	65
	Int	14	fix	16.5	11.3	4.3	7.9	0.75	0.88	0.71	0.73	66
			relax	15.6	10.4	4.1	7.7	0.77	0.85	0.71	0.67	71
		15	fix	16.2	10.0	4.2	8.0	0.74	0.87	0.43	0.70	73
			relax	15.1	9.2	3.9	8.0	0.79	0.90	0.71	0.76	53
	Big	14	fix	15.3	9.9	3.8	7.6	0.69	0.84	0.43	0.67	79
			relax	12.8	10.0	3.3	7.2	0.88	0.93	0.71	0.82	43
TPSS/TZ sp	Min	14	fix	16.9	11.7	4.5	8.5	0.80	0.87	0.71	0.67	85
			relax	16.5	11.3	4.6	8.2	0.81	0.91	0.71	0.73	60
	Int	14	fix	16.4	11.1	4.5	8.1	0.74	0.85	0.71	0.67	93
			relax	16.4	10.1	4.3	7.9	0.78	0.88	0.71	0.73	60
		15	fix	16.0	9.5	4.0	7.8	0.74	0.85	0.43	0.67	76
			relax	14.6	8.3	4.1	7.2	0.76	0.88	0.71	0.73	54
TPSS/cc	Min	14	fix	16.7	11.6	4.5	8.7	0.80	0.87	0.71	0.67	85
B3LYP/SV sp	Min	14	fix	17.7	11.9	4.4	9.1	0.78	0.87	0.71	0.67	90
			relax	15.4	10.0	3.9	7.7	0.78	0.88	0.71	0.70	57
	Int	14	fix	17.0	10.8	4.3	8.4	0.77	0.88	0.71	0.73	68
			relax	15.4	9.3	4.1	7.3	0.78	0.88	0.71	0.73	55
		15	fix	16.0	9.7	4.1	7.9	0.73	0.87	0.43	0.70	69
			relax	14.9	8.1	3.8	7.0	0.76	0.88	0.71	0.73	52
	Big	14	fix	15.9	9.4	3.7	8.1	0.67	0.84	0.43	0.67	85
			relax	15.0	7.9	3.6	7.7	0.69	0.82	0.71	0.64	80
B3LYP/TZ sp	Min	14	fix	16.9	11.5	4.4	8.3	0.79	0.87	0.71	0.67	82
			relax	16.5	11.0	4.4	8.0	0.81	0.89	0.71	0.70	64

a The last column shows our ranking comparing all the 113 tested methods. QMS is the size of the QM system (Min, Int, or Big). FF is the force field, FF14SB or FF15IPQ. Surr marks whether the surroundings were fixed or relaxed. Method reports the QM method (TPSS or B3LYP), the basis set (def2-SV(P), def2-TZVPD, or aug-cc-pVTZ, abbreviated SV, TZ, and cc) and whether the redox calculation was performed on a geometry optimized with the same method (opt) or not (sp). Only the TPSS/SV redox calculations were performed on a geometry optimized with the same method, whereas the other redox calculations were based on TPSS/SV structures using the same QMS, FF, and surroundings. For all QM/MM calculations, MSE is the negative of MAD.

From these results, it can be seen that all the QM/MM variants give poor results. The best result (ranking number 43 compared to the QTCP and QM + COSMO potentials) is obtained with TPSS-D3/def2-SV(P), FF14SB, the big QM system, and relaxed surroundings. Yet, QM/MM gives the best τ_66_ values among all methods (0.64–0.82, compared to −0.58 to 0.76 for the other methods). Likewise, ρ of the QM/MM methods is better than for most of the other methods (0.82–0.93, compared to −0.76 to 0.90). The *R*^2^ values are also good (0.67–0.88, compared to 0.01–0.85). On the other hand, the ranges of the QM/MM calculations are very poor, showing an overestimation of the experimental range by a factor of 13–18. Consequently, the MAD (7.9–12.3 V) and MADtr (3.3–4.6 V) values are also poor, and the MAXtr value is 7.0–9.2 V. In fact, the calculated potentials are always too negative. These results reflect that QM/MM does not take proper account of the long-range solvation effects (because the whole or the outer part of the MM system is fixed) and that only the QM system is polarizable. Allowing the closest 6 Å of the surroundings to relax in general improves the range, MAD, and MADtr measures, but not very much. Still, the ranking improves in all except one case. Changing the force field from FF14SB to FF15IPQ improves the ranking for four of the six cases. On the other hand, changing the QM method from TPSS to B3LYP, increasing the size of the QM system or improving the basis set, gives unclear trends.

### QTCP Results

QTCP calculations are much more time-consuming than the other methods because they involve FEP calculations based on MD simulations. Therefore, we tested only three variants: one with the minimal QM system and two with the intermediate QM system and different force fields. Each of them was based on QM/MM structures obtained with fixed surroundings (but all atoms in the surroundings move freely in the MD simulations). For each of them, we tested three different ways to treat the long-range electrostatics (Born, Ewald, or GB) and included or excluded solvent-accessible charged groups. We also tested to neutralize all charged residues on the surface of the studied proteins, before doing the QM/MM and MD simulations for the minimal QM systems. The calculated redox potentials from the 24 variants of QTCP are shown in Table S3, and the quality measures from the QTCP calculations are collected in Table 3.

**Table 3:** Quality Measures (Range, MSE, MAD, MADtr, and MAXtr in V; *R*^2^, ρ, τ_7_, and τ_66_) of the QTCP Calculations^a^

QMS	FF	LR	SE	Range	MSE	MAD	MADtr	MAXtr	*R*^2^	ρ	τ_7_	τ_66_	rank
Min	14	B/O	Exc	7.0	–7.3	7.3	1.4	3.8	0.81	0.88	0.71	0.70	38
			Inc	10.0	–0.7	3.2	3.0	6.3	0.32	–0.61	–0.14	–0.45	109
		GB	Exc	7.4	–2.4	3.0	1.5	5.8	0.03	0.00	–0.14	0.06	105
			Inc	4.8	–3.5	3.5	0.8	2.6	0.35	0.29	0.71	0.21	96
		Ew	Exc	3.4	–3.6	3.6	0.5	1.7	0.30	0.43	1.00	0.33	89
			Inc	6.6	–0.8	2.4	2.3	4.2	0.46	–0.70	–0.43	–0.55	98
Int	14	B/O	Exc	10.9	–4.8	5.3	2.0	8.2	0.08	0.57	0.43	0.42	102
			Inc	13.6	1.1	2.7	2.8	10.2	0.31	–0.73	–0.14	–0.55	111
		GB	Exc	11.0	–0.4	2.7	2.5	8.4	0.02	–0.06	0.14	–0.06	111
			Inc	10.5	–1.2	2.5	1.6	8.8	0.09	–0.38	0.14	–0.30	110
		Ew	Exc	10.5	–1.1	2.3	1.5	8.7	0.01	0.33	0.43	0.24	107
			Inc	13.2	1.5	2.6	2.6	10.3	0.29	–0.76	–0.43	–0.58	113
	15	B/O	Exc	9.5	–3.5	3.8	1.5	5.2	0.08	0.44	0.43	0.42	97
			Inc	10.9	2.7	3.5	2.8	6.8	0.50	–0.71	–0.14	–0.48	106
		GB	Exc	8.6	0.4	1.8	1.8	6.2	0.22	–0.52	–0.14	–0.30	102
			Inc	7.8	0.3	1.6	1.6	5.5	0.22	–0.45	–0.14	–0.24	100
		Ew	Exc	8.0	0.4	1.4	1.5	5.6	0.05	–0.15	0.14	0.00	99
			Inc	10.7	3.0	3.2	2.5	7.1	0.45	–0.72	–0.43	–0.48	108
Min^b^	14	B/O	Exc	3.9	–4.8	4.8	0.6	2.1	0.51	0.61	1.00	0.42	70
			Inc	5.9	–4.1	4.1	1.1	4.4	0.02	0.19	0.14	0.15	104
		GB	Exc	2.4	–3.0	3.0	0.6	1.3	0.18	0.34	0.43	0.21	75
			Inc	2.6	–3.0	3.0	0.6	1.6	0.16	0.22	0.43	0.18	92
		Ew	Exc	3.3	–3.6	3.6	0.5	1.7	0.36	0.48	1.00	0.36	78
			Inc	4.6	–3.1	3.1	0.9	3.3	0.03	0.17	0.14	0.15	101

a The last row shows the final ranking among all the 113 tested methods. QMS is the size of the QM system. FF is the force field, FF14SB or FF15IPQ. LR is the long-range corrections, Born/Onsager, generalized Born or Ewald. SE is the treatment of solvent-exposed charged residues, excluded or included. All QM calculations were performed at the TPSS/def2-SV(P) level of theory, and the QM/MM geometry optimizations were performed with fixed surroundings.

b Calculations with all solvent-exposed charged groups neutralized before the MD simulations.

Somewhat unexpectedly, all the 24 variants give a quite large range of the calculated potentials, 2–14 V, that is, intermediate between those of QM/MM and QM + COSMO. On the other hand, the MAD values are smaller, 1.4–7.3 V, reflecting that some calculated redox potentials are positive. For one of the variants (the Min QM system with Born solvation and solvent-exposed charges excluded), *R*^2^, ρ, and τ are quite good (*R*^2^ = 0.81, ρ = 0.88, and τ_66_ = 0.70). However, MADtr and MAXtr are quite poor (1.4 and 3.8 V), giving it a total rank of 38. For the other QTCP variants, the results are worse and the ranking is poor, 70–113.

Excluding the solvent-exposed charged groups gives a better ranking than including them in 75% of the calculations. When they are excluded, Born/Onsage treatment of the long-range electrostatics gives the best results, whereas generalized Born calculations give the best results when they are included. The FF15IPQ force field always gives a better ranking than the FF14SB force field. Neutralizing the surface charges already in the QM/MM and MD simulations improves the results for four of the six variants. It improves the range to 2.4–5.9 V and MADtr to 0.5–1.1 V.

A similar method (FEP calculations with combined QM/MM potentials) has been used to evaluate the reduction potential of FAD in cholesterol oxidase, and the obtained accuracy relative to the experimental result was 0.8 V,^141^ that is, similar to the MADtr value of the best QTCP variants.

### QM + COSMO Redox Potentials

Next, we considered 64 variants of QM + COSMO calculations, in which we took the QM system from the QM/MM-optimized structures (always optimized by TPSS/def2-SV(P)) and performed a single-point QM calculation in a COSMO continuum solvent. The variants involve three different dielectric constants (ε = 4, 20, or 80), systems of the three sizes, two DFT methods, three different basis sets, two different force fields (used in the QM/MM geometry optimizations), and whether the surroundings were fixed or relaxed in the QM/MM optimizations. We also tested to use QM/MM structures optimized with a smaller QM system than the one used in the COSMO redox calculations. The redox potentials from the QM + COSMO calculations are listed in Tables S4 and S5 in the Supporting Information, and the quality measures are collected in Table 4.

**Table 4:** Quality Measures (Range, MSE, MAD, MADtr, and MAXtr in *V*; *R*^2^, ρ, τ_7_, and τ_66_) of the QM + COSMO Calculations^a^

method	Surr	QMS	FF	ε	Range	MSE	MAD	MADtr	MAXtr	*R*^2^	ρ	τ_7_	τ_66_	rank
TPSS/SV	fix	Min	14	4	4.46	–3.10	3.10	0.81	2.46	0.64	0.76	0.14	0.58	51
				20	2.58	–1.52	1.52	0.55	1.04	0.51	0.72	0.43	0.52	37
				80	2.18	–1.19	1.19	0.52	0.89	0.45	0.67	0.43	0.45	32
		Int	14	4	2.62	–1.74	1.74	0.39	1.02	0.78	0.86	0.43	0.73	16
				20	1.69	–0.77	0.77	0.29	0.58	0.73	0.79	0.71	0.64	10
				80	1.59	–0.56	0.56	0.27	0.52	0.70	0.78	0.71	0.61	8
			15	4	2.69	–1.76	1.76	0.42	1.11	0.75	0.85	0.14	0.70	22
				20	1.70	–0.78	0.78	0.31	0.62	0.72	0.76	0.43	0.61	13
				80	1.59	–0.58	0.58	0.29	0.57	0.67	0.75	0.43	0.58	12
		Big	14	4	2.50	–1.81	1.81	0.54	1.15	0.73	0.81	0.43	0.61	27
				20	1.40	–0.83	0.83	0.23	0.46	0.78	0.87	0.71	0.70	4
				80	1.31	–0.62	0.62	0.17	0.44	0.74	0.85	0.71	0.67	2
				∞	1.27	–0.55	0.55	0.17	0.44	0.69	0.85	0.71	0.67	1
TPSS/TZ	fix	Min	14	4	4.19	–2.89	2.89	0.81	2.10	0.58	0.66	–0.43	0.48	56
				20	2.32	–1.17	1.17	0.57	1.21	0.07	0.19	–0.14	0.12	52
				80	2.16	–0.65	0.81	0.56	1.25	0.21	0.50	–0.14	0.33	44
		Int	14	4	5.44	–1.33	1.56	1.09	2.54	0.37	0.50	–0.14	0.39	62
				20	4.18	–0.44	0.93	0.82	2.17	0.05	0.13	0.14	0.03	64
				80	4.12	–0.36	0.97	0.91	2.10	0.07	0.15	–0.14	0.09	63
TPSS/cc	fix	Min	14	4	3.04	–2.68	2.68	0.67	1.53	0.60	0.71	0.14	0.55	50
				20	2.16	–1.09	1.09	0.48	1.08	0.31	0.62	0.14	0.45	36
				80	1.97	–0.75	0.84	0.49	1.09	0.22	0.50	0.14	0.33	42
TPSS/SV	relax	Min	14	4	3.47	–3.22	3.22	0.69	1.48	0.62	0.71	0.43	0.48	53
				20	2.56	–1.61	1.61	0.51	1.03	0.39	0.66	0.14	0.45	41
				80	2.36	–1.27	1.27	0.50	0.94	0.30	0.60	0.14	0.42	38
		Int	14	4	2.64	–1.73	1.73	0.42	1.18	0.76	0.81	0.14	0.64	23
				20	1.64	–0.76	0.76	0.30	0.52	0.74	0.78	0.71	0.64	9
				80	1.55	–0.56	0.56	0.28	0.47	0.69	0.74	0.71	0.58	11
			15	4	2.69	–1.80	1.80	0.46	0.99	0.71	0.81	0.14	0.64	24
				20	1.78	–0.81	0.81	0.34	0.68	0.67	0.75	0.43	0.58	15
				80	1.64	–0.60	0.61	0.32	0.64	0.62	0.73	0.43	0.55	14
		Big	14	4	2.53	–1.84	1.84	0.57	0.97	0.78	0.86	0.14	0.73	19
				20	1.70	–0.85	0.85	0.24	0.79	0.80	0.90	0.43	0.73	7
				80	1.53	–0.64	0.64	0.22	0.75	0.74	0.89	0.43	0.70	5
B3LYP/SV	fix	Min	14	4	3.00	–2.90	2.90	0.70	1.40	0.40	0.55	–0.14	0.36	57
				20	2.20	–1.29	1.32	0.58	1.44	0.09	0.38	–0.14	0.24	54
				80	2.19	–0.95	1.09	0.60	1.52	0.04	0.16	–0.14	0.06	55
		Int	14	4	2.87	–1.36	1.36	0.69	1.00	0.72	0.82	1.00	0.73	26
				20	2.84	–0.39	0.70	0.59	1.09	0.52	0.72	1.00	0.64	31
				80	2.83	–0.19	0.64	0.58	1.17	0.46	0.69	1.00	0.58	34
			15	4	3.23	–1.49	1.49	0.62	1.59	0.53	0.75	0.43	0.58	46
				20	4.28	–0.37	0.87	0.70	2.90	0.08	0.45	0.14	0.39	60
				80	2.53	–0.32	0.62	0.50	1.33	0.28	0.48	0.43	0.39	40
		Big	14	4	3.10	–1.42	1.42	0.58	1.47	0.51	0.69	–0.14	0.42	49
				20	1.97	–0.41	0.63	0.43	0.99	0.33	0.55	0.14	0.39	29
				80	1.87	–0.21	0.54	0.43	0.99	0.25	0.50	0.43	0.36	30
B3LYP/SV	relax	Min	14	4	3.43	–2.89	2.89	0.76	1.62	0.37	0.56	–0.14	0.36	61
				20	2.75	–1.28	1.34	0.64	1.56	0.09	0.39	–0.14	0.24	57
				80	2.73	–0.94	1.13	0.66	1.64	0.05	0.24	–0.14	0.12	59
		Int	14	4	2.83	–1.35	1.35	0.72	1.05	0.71	0.81	0.71	0.70	28
				20	2.82	–0.39	0.71	0.62	1.04	0.53	0.67	0.71	0.55	35
				80	2.82	–0.19	0.65	0.60	1.12	0.47	0.65	0.71	0.52	39
			15	4	3.17	–1.55	1.55	0.65	1.59	0.50	0.75	0.43	0.58	48
				20	2.55	–0.56	0.75	0.56	1.37	0.32	0.46	0.14	0.36	45
				80	2.56	–0.35	0.66	0.53	1.33	0.27	0.44	0.14	0.33	47
		Big	14	4	2.76	–1.43	1.43	0.59	1.14	0.68	0.77	0.14	0.58	33
				20	2.19	0.42	0.53	0.35	0.97	0.54	0.66	0.43	0.52	21
				80	2.07	–0.22	0.43	0.36	0.93	0.46	0.55	0.43	0.42	25
TPSS/SV	fix	Min	14	4	2.96	–2.04	2.04	0.61	1.25	0.69	0.77	0.43	0.55	43
Big QM				20	1.72	–1.04	1.04	0.36	0.76	0.63	0.76	0.71	0.61	16
				80	1.52	–0.82	0.82	0.33	0.72	0.57	0.73	0.71	0.55	16
		Int	14	4	2.73	–2.02	2.02	0.56	1.06	0.83	0.90	0.43	0.73	20
				20	1.82	–1.02	1.02	0.25	0.57	0.85	0.90	0.71	0.73	6
				80	1.67	–0.81	0.81	0.21	0.53	0.79	0.90	0.71	0.76	2

a The last row shows our ranking (involving only these methods). The Method column describes the QM method (TPSS or B3LYP) and the basis set (def2-SV(P), def2-TZVPD, or aug-cc-pVTZ, abbreviated SV, TZ, and cc, respectively) used in the QM + COSMO calculations, whereas QMS is the size of the QM system. All QM + COSMO calculations were single-point energy calculations on structures optimized with QM/MM and the TPSS-D3/def2-SV(P) level of theory. Surr marks whether the surroundings were fixed or relaxed, and FF is the force field used in the QM/MM optimizations, FF14SB or FF15IPQ. The results in the six last rows were obtained with the big QM system based on QM/MM structures optimized with the Min or Int QM systems.

As already indicated, most of the QM + COSMO redox potentials are appreciably better than those of the QM/MM and QTCP redox potentials according to our total ranking score (including the 37 best ranking variants). Therefore, we will not include the QM/MM and QTCP potentials when ranking the QM + COSMO results.

Somewhat unexpectedly, calculations with the larger def2-TZVPD basis set give worse results than those with the smaller basis set, ranking 44–64. This is connected with a large range of the calculated results, 2.2–5.4 V, especially for the intermediate QM system. However, the *R*^2^, ρ, and τ correlations are also rather poor with the larger basis set. We also tested another large basis set, aug-cc-pVTZ, which gives slightly better results (rankings of 36–50), similar to those of the def2-SV(P) basis set. Sundararajan et al. also found no improvement with larger basis sets for rubredoxin models.^51^

Calculations with the minimal QM system give relatively poor results, with ranks of 32–61 among the QM + COSMO calculations. Calculations with B3LYP-D3 also give worse results than TPSS-D3, providing ranks of 21–60 for the intermediate and large QM systems. In most cases (75%), calculations with fixed surroundings give better results than when the surroundings were relaxed, but the difference is rather small.

Thus, the calculations with the intermediate and big QM systems with TPSS/def2-SV(P) give the best results (ranks 1–27). The two force fields (for the QM/MM structures) give nearly identical results, showing that the method is not sensitive to the structures used. In fact, the individual calculated absolute redox potentials differ on average by only 0.05 V. Therefore, we will restrict the discussion to the structures obtained with the FF14SB force field, which always gives a slightly better ranking.

For the remaining QM + COSMO calculations, calculations with the smallest dielectric constant (ε = 4) give worse results than the other two dielectric constants (in terms of ranking; 16–27). However, the trends are varying because they involve opposing effects. In all cases, the range, MAD, MADtr, and MAXtr decrease (i.e., improve) when ε is increased. On the other hand, *R*^2^, ρ, and τ typically show the opposite trend, with deteriorated results as ε is increased (not always for the big QM system). Thus, the user could, in principle, fine-tune the method depending on whether a good correlation or low MADtr and MAXtr is preferred.

According to our ranking, the best results are obtained with the big QM system and ε = 80. With fixed surroundings, it gives the best MADtr (0.17 V), range (1.3 V), and MAXtr (0.44 V). The MAD, ρ, *R*^2^, and τ_66_ are more mediocre (0.6 V, 0.85, 0.74, and 0.67), ranking 10–13. All redox potentials are negative, less than −0.15 V; thus, the systematic error (MSE) is the negative of the MAD, −0.62 V. In fact, the results can be slightly improved by using an infinite dielectric constant, giving a MAD of 0.55 V, but a slightly lower *R*^2^ = 0.69. The corresponding calculation with ε = 20 gives slightly worse results with MADtr = 0.23 V, MAXtr = 0.46 V, and range = 1.4 V, but it gives slightly better *R*^2^, ρ, and τ_66_, 0.78, 0.87, and 0.70. The corresponding calculations with relaxed surroundings rank 7 and 5 for ε = 20 and 80, respectively. Likewise, the calculations with the intermediate QM system rank 8–11, whereas those with FF15IPQ rank 12–15.

It is notable that most of the best methods show rather large systematic errors, for example, MSE = −MAD = −0.5 to −0.9 V for the best QM-COSMO methods discussed so far. This is of course alarming, illustrating the importance of calibration calculations to obtain more reasonable relative errors (MADtr = 0.2–0.3 V). The results in Table 4 show that MSE and MAD improve with the dielectric constant. They are also worst for the minimal QM system, and the MSE is negative for the QM + COSMO methods. However, in variance to our ranking of the methods, MSE always improves for B3LYP compared to TPSS. In fact, the lowest MSE, −0.2 V, is obtained with B3LYP, ε = 80, the Int or Big QM systems with FF14SB, and both fixed or relaxed surroundings. However, with B3LYP and ε = 20 or 80, the calculations no longer systematically underestimate the redox potentials, so the MADs are larger. Still, the smallest MAD, 0.43 V, is obtained with B3LYP (the Big QM system, ε = 80 and relaxed surroundings). This shows that the absolute redox potentials are largely coupled to the DFT method, and the errors are systematic. It is possible that if we had tested more DFT methods, we might find a method with MSE and MAD closer to zero. However, as the systematic error can be compensated by calibration calculations and as relative redox potentials are normally more interesting than absolute potentials, we have not concentrated on optimizing the absolute potentials in this study.

Likewise, it can be seen from Table 4 that the MSE is also always improved when the basis set is increased from def2-SV(P) to def2-TZVPD (the aug-cc-pVTZ basis set also gives better results than def2-SV(P) but worse than def2-TZVPD). This again illustrates that the absolute redox potentials depend strongly on the QM method, and they actually improve when the basis set is improved. However, as mentioned above, the other quality measures deteriorate with the larger basis set. This shows that for accurate relative potentials, it is more important that the systematic errors are consistent and predictable, which apparently is the case for the smaller basis sets and TPSS.

Since optimizing the structures with the largest QM systems is quite time-consuming, we tried to do QM + COSMO calculations with the big QM system based on QM/MM structures optimized with the minimal or intermediate systems (last six rows of Table 4). The results based on the minimal QM system are rather poor, ranking 16–43, but appreciably better than the QM + COSMO results on the minimal QM system (ranking 32–51). However, the results based on the QM/MM calculation on the intermediate QM system give results that are comparable with the best QM + COSMO results with the big QM system. The calculation with ε = 80 gives the second best result in this investigation with MADtr = 0.21 V, range = 1.7 V, MSE = −0.8 V, MAD = 0.8 V, MAXtr = 0.5 V, *R*^2^ = 0.79, ρ = 0.90, and τ_66_ = 0.76. The first five are slightly worse than those obtained with QM/MM structures from the large QM system, but the latter three are better and the total ranking is the same. Thus, this seems to be a good approach to reduce the cost of the calculations.

**Figure 2:**
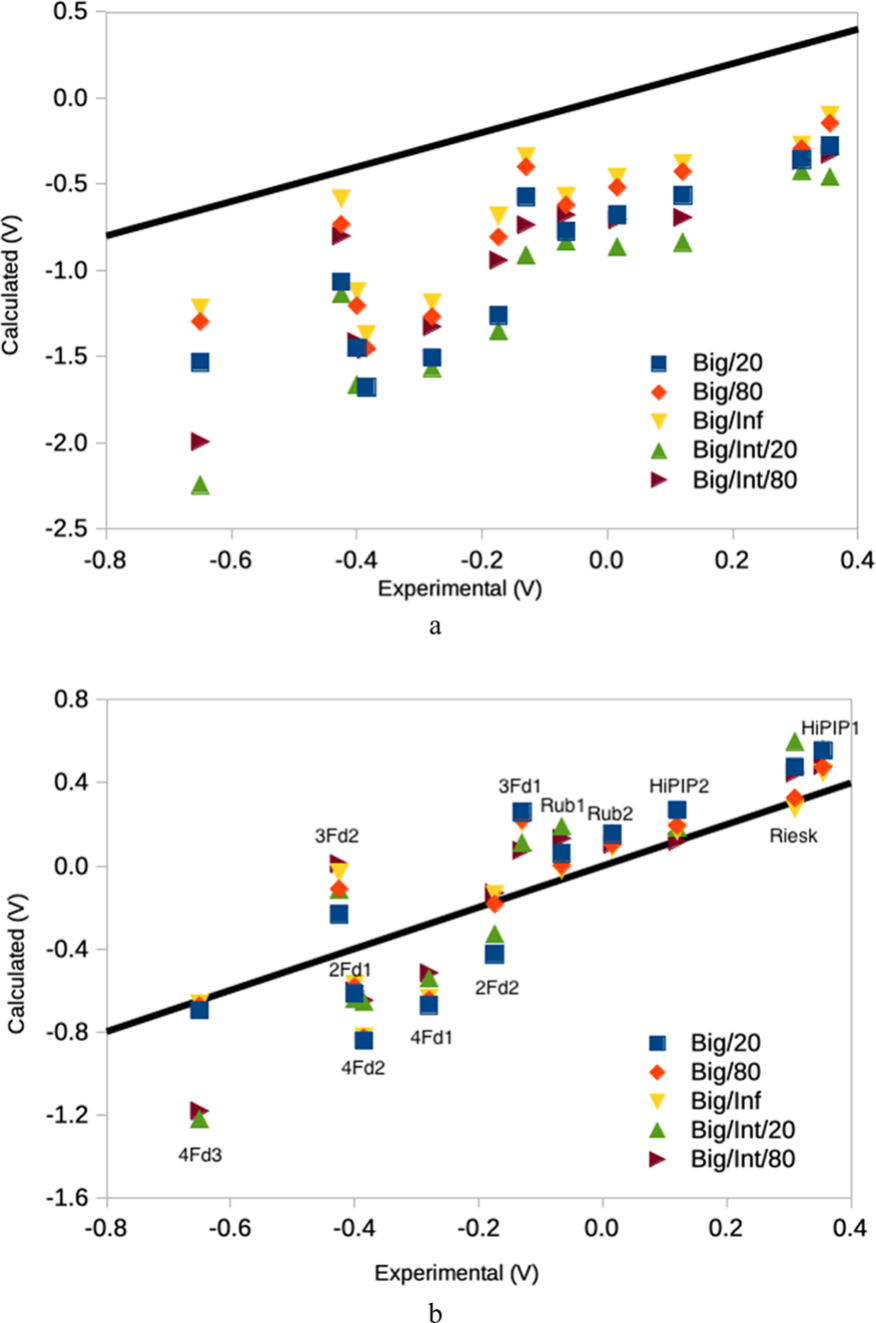
Performance of some of the best QM + COSMO methods (all with TPSS-D3/def2-SV(P) and fixed surroundings) for the (a) absolute redox potentials or the (b) potentials translated by the systematic error (MSE). The methods are denoted after the size of the QM system and the dielectric constant. Big/Int uses the big QM system for the QM + COSMO calculations based on the Int QM/MM structures.

Figure 2 illustrates the performance of five of the best methods. It can be seen that even after removing the systematic errors (−0.5 to −1.0 V), there remain clear differences between the various types of sites: the 4Fd and 2Fd sites are below the calibration line, whereas the other sites are above the line. This indicates that the results will be better if only sites of the same type are considered. Consequently, all pairs of potentials from the same type of iron–sulfur clusters are ranked correctly, except one for all these methods (i.e., τ_7_ = 0.71).

### Discriminatory Power

Finally, we examined the discriminatory power of some of the methods by studying whether they can predict the correct redox couple for the 4Fd1 site. Thus, we calculated the redox potentials for four redox couples: Fe_4_^II^Fe_0_^III^/Fe_3_^II^Fe_1_^III^, Fe_3_^II^Fe_1_^III^/Fe_2_^II^Fe_2_^III^, Fe_2_^II^Fe_2_^III^/Fe_1_^II^Fe_3_^III^, and Fe_1_^II^Fe_3_^III^/Fe_0_^II^Fe_4_^III^. We subtracted the systematic error obtained in this study for the 12 different iron–sulfur clusters (similar results were obtained if the 4Fd1 site was excluded for this calculation of the MSE) and compared the calculated redox potential to the experimental one (−0.28 V).^93^ We wanted to decide which methods can identify the correct redox couple within the maximum error of the method (MAXtr from this study).

The results are shown in Table 5. It can be seen that for the two tested QM/MM methods, the MAXtr is so large (9 V) that all the four redox couples give calculated redox potentials that agree with the experimental one within MAXtr. However, for all the tested QM + COSMO methods, except one, the calculations point out the correct Fe_3_^II^Fe_1_^III^/Fe_2_^II^Fe_2_^III^ redox couple as the only one that reproduces the experimental data within MAXtr. The other couples give errors of 1.2–4.1 V. Thus, the present calibration line (MSE and MAXtr) would allow us to determine the correct redox couple, even if the accuracy in absolute terms is rather poor.

**Table 5:** Redox Potentials Calculated for the Four Possible Redox Couples of the 4Fd1 Site: Fe_4_^II^Fe_0_^III^/Fe_3_^II^Fe_1_^III^, Fe_3_^II^Fe_1_^III^/Fe_2_^II^Fe_2_^III^, Fe_2_^II^Fe_2_^III^/Fe_1_^II^Fe_3_^III^, and Fe_1_^II^Fe_3_^III^/Fe_0_^II^Fe_4_^III^ (Called 4/3, 3/2, 2/1, and 1/0 in the Table) for 20 Different Methods (All with TPSS-D3/def2-SV(P))^a^

QM system	force field	surroundings	ε	MSE	MAXtr	4/3	3/2	2/1	1/0
QM/MM									
Min	FF14SB	fix		–12.3	9.2	**–8.6**	**–4.9**	**–0.8**	**2.7**
		relax		–11.5	8.6	**–7.0**	**–4.4**	**–0.9**	**2.3**
QM-COSMO									
Min	FF14SB	fix	4	–3.1	2.5	–2.9	**–0.9**	**1.4**	3.1
			20	–1.5	1.0	–1.8	**–0.6**	1.0	1.9
			80	–1.2	0.9	–1.6	**–0.5**	0.9	1.7
		relax	4	–3.2	1.5	–2.7	**–0.9**	1.5	3.1
			20	–1.6	1.0	–1.7	**–0.6**	1.1	1.9
			80	–1.3	0.9	–1.5	**–0.5**	1.0	1.7
Int	FF14SB	fix	4	–1.7	1.0	–2.6	**–0.8**	1.4	2.8
			20	–0.8	0.6	–1.8	**–0.6**	1.1	2.0
			80	–0.6	0.5	–1.6	**–0.5**	1.0	1.8
		relax	4	–1.7	1.2	–2.8	**–0.8**	1.4	2.8
			20	–0.8	0.5	–2.0	**–0.6**	1.1	2.0
			80	–0.6	0.5	–1.8	**–0.5**	1.0	1.8
	FF15IPQ	fix	4	–1.8	1.1	–2.6	**–1.1**	1.6	2.8
			20	–0.8	0.6	–1.8	**–0.9**	1.2	2.1
			80	–0.6	0.6	–1.6	**–0.9**	1.2	1.9
		relax	4	–1.8	1.0	–3.0	**–1.1**	1.7	2.9
			20	–0.8	0.7	–2.2	**–0.9**	1.3	2.1
			80	–0.6	0.6	–2.1	**–0.9**	1.2	1.9

a The table shows the systematic error (MSE) and the maximum error (MAXtr) for each method (from Table 2 orTable 4), as well as the calculated redox potential for the four redox couples, corrected by MSE, all in V. Results that agree with the experimental redox potential (−0.28 V) within MAXtr are highlighted in bold face.

### Time Consumption

Finally, we discuss the time consumption of the various methods. The timings are shown in Tables S19–S21 in the Supporting Information, presented as the sum over all 12 systems (to reduce the dependence of the number of wave function and geometry optimization iterations). All methods are based on the TPSS/def2-SV(P) QM/MM structures. Optimizations with the intermediate QM system were ∼13 times more time-consuming than those with the minimal QM system and those with the large QM system were ∼3 times more expensive than those with the intermediate system. Optimizations with relaxed surroundings were 18–33% more time-consuming than the corresponding optimizations with fixed surroundings.

Single-point QM calculations with the def2-TZVPD basis set took only 19–52% of the corresponding time for the geometry optimization with the def2-SV(P) basis set. Calculations with B3LYP were 10–13 times more expensive than the corresponding TPSS calculations. However, single-point B3LYP energy calculations with the def2-SV(P) basis set took only 1–5% of the corresponding TPSS/def2-SV(P) geometry optimizations. Consequently, the QM + COSMO calculations were fast, taking only 1–3% of the corresponding time for the corresponding QM/MM geometry optimization. Calculations with ε = 20 and 80 were approximately 50% more time-consuming than the calculations with ε = 4. The total timing for the preferred QM + COSMO(ε = 80)/Big/FF14/Fix calculations is ∼95 CPU hours on 20 cores for all 12 proteins, which is not prohibitive (the time needed for the setup of the proteins is larger than that).

The QTCP calculations are quite time-consuming, 4–26 times more expensive than the QM/MM geometry optimizations for the minimal QM system and up to 3 times more time-consuming for the intermediate QM systems. The Ewald postprocessing is ∼60 times faster than the GB simulations, but the QTCP time is dominated by the MM → QM/MM perturbation, especially for the intermediate QM system.

## Conclusions

In this study, we have evaluated how well variants of QM/MM, QTCP, and QM + COSMO calculations estimate the redox potentials of 12 iron–sulfur sites of the most common types observed in proteins. We evaluated the stability of the estimates by performing variations of the methods, in terms of the DFT method, the basis set, the size of the QM system, the force field, the dielectric constant, long-range corrections, and whether the surroundings are optimized or not.

Using seven quality criteria, viz., the range, MAD, MADtr, MAXtr, *R*^2^, ρ, and τ_66_, we show that QM + COSMO gives much better results than the raw QM/MM energies and the QTCP calculations. Among the QM + COSMO calculations, using a big QM system (∼300 atoms) is important to obtain accurate results. However, equally good results can be obtained by using the big QM system in a single-point calculation based on a QM/MM structure obtained with an intermediate-sized QM system (∼150 atoms). For the current systems, TPSS gives better results than B3LYP, and no advantage of increasing the basis set from def2-SV(P) to def2-TZVPD or aug-cc-pVTZ is seen. Likewise, no advantage of relaxing the surroundings is observed. The force field used for the QM/MM structures has a rather small influence on the potentials calculated with QM + COSMO, and FF14SB gives slightly better results than FF15IPQ, whereas the opposite was observed for the QM/MM potentials. Clearly, a rather large dielectric constant is needed for accurate results. However, ε = 80 gives better energy-based criteria but a worse ranking than ε = 20. Further increasing the dielectric constant to infinity continues this trend, but the change in the actual potentials is small (0.07 V on average). On the whole, we recommend using the big QM system and a dielectric constant of 80, which gives MADtr = 0.17 V and MAXtr = 0.44 V.

Such an accuracy is comparable to what was obtained with a similar QM + continuum solvation approach for six blue copper proteins (MADtr = 0.19 V), although it did not use QM/MM structures.^127^ At first, the obtained accuracy may seem somewhat disappointing, making it hard to provide useful predictions, for example, for the effect of site-directed mutations. However, with the average and maximum errors from this study, it is possible to answer more general questions, for example, regarding the employed oxidation-state levels and the charge state of the cluster, as was shown by the calculations for the four redox-state levels for 4Fd1 in Table 5. This can be very useful for the study of more complicated iron–sulfur clusters. We are currently working with such a study of P and FeMo clusters in nitrogenase.

Many factors contribute to differences between experimentally measured and calculated redox potentials. The calculated potentials are dominated by two terms, the electronic ionization potential and the solvation energy. The former term strongly depends on the QM method. It can be benchmarked toward experimental (gas-phase) data or high-level QM methods.^30,142^ Unfortunately, no such data are available for iron–sulfur clusters. For Fe^2+/3+^ models with water molecules and sometimes one protein-like ligand (e.g., CH_3_S^–^), hybrid functionals with a large amount of Hartree–Fock exchange give the best results, with MADs of 0.08 eV and maximum errors of 0.2 eV (0.11 and 0.27 eV for B3LYP).^30^ However, it is likely that methods with a lower amount of Hartree–Fock exchange perform better for iron–sulfur clusters with their larger contribution of static correlation.^143^

The solvation energy is much harder to benchmark because it depends on the detailed modeling of the surroundings. Here, the comparison needs to be done to experimental redox potentials of proteins. Naturally, experimentally measured redox potentials also have limited accuracy and precision. For simple metal complexes in solution, the accuracy of the calculations has reached the level (by benchmark studies) that errors in experimental studies may be identified.^17^ Moreover, it has been shown that the experimental potentials depend significantly on the electrode and the electrolyte (besides the obvious dependence on the solvent).^29^ It was argued that both experiments and calculations should be compared to a reference potential measured at the same conditions or calculated with the same methods and for metals from the same row in the periodic table. For proteins, the calculations have not yet reached this accuracy, and measurements of closely related proteins from other organisms are often available, giving confidence to the experimental data.^31^

When comparing experimental and computational data, it should always be asked if they measure the same thing. The calculations are typically based on crystal structures obtained at conditions quite different (e.g., cryogenic temperatures and in crystals) from those used in the redox experiments. Fortunately, crystal structures and solution structures are typically closely similar. On the other hand, experimental redox potentials are often sensitive to the measuring conditions, for example, temperature, pH, and ionic strength. In the calculations, these are modeled by the dielectric constant, protonation states of the protein residues, and counterions. Naturally, the calculations strongly depend on the protonation state selected for the QM system, and in particular, the iron–sulfur cluster itself. For the clusters in this study, no change in the protonation of the cluster is expected at neutral pH.^63^ It is the hope that the present calibration would allow the detection of direct protonation of the cluster. For the protonation of the surrounding protein, we employ simple manual and empirical methods to decide the protonation state at pH 7. It has been shown that simpler properties, such as ligand-binding affinities, are relatively insensitive to the detailed protonation states, except for residues very close to the active site.^144^ However, it is likely that redox calculations are more sensitive to protonation states, owing to the change in the net charge of the iron–sulfur cluster upon reduction. In this respect, the QM + COSMO calculations are much less sensitive to the details of the protein setup because a smaller part of the protein is explicitly included in the calculations.

It should be noted that our quality criteria favor methods that give accurate relative redox potentials. If the absolute potentials are of main interest, B3LYP gives better results than TPSS, and increasing the basis sets from def2-SV(P) to def2-TZVPD improves the results, more reflecting the expected performance of DFT methods. On the other hand, accurate relative redox potentials are obtained for methods that give more consistent and predictable errors.

At first, it may seem unexpected that the QM + COSMO calculations, which include only a minor part of the protein in the calculations, give better results than both QM/MM and QTCP calculations, which include an explicit account of the full proteins and a considerable amount of the surrounding solvent. This most likely reflects the importance of correctly modeling the dielectric relaxation of the surroundings to the change in the net charge of redox-active sites. In the QM + COSMO calculations, the QM calculations automatically include the electronic relaxation, and the COSMO model provides a proper relaxation of the surroundings, modeled by a continuum solvent with a large dielectric constant. Moreover, both relaxations affect each other, and this interdependence is taken into account in a proper self-consistent manner. In the QM/MM calculations, only the electronic relaxation of the QM system is properly treated, whereas the surroundings are treated with a fixed-charge model, which cannot account for electronic relaxation (polarization). This often gives rise to overpolarization of the QM system.^5^ The relaxed QM/MM calculations allow some minor relaxation of the surroundings, but the effect is quite minor because it is only a local minimization and the relaxed system is quite small, not including most of the solvent. In the QTCP calculations, a more proper relaxation of the surroundings is taken into account, involving all atoms, except the QM system, but still with a fixed-charge MM model. Moreover, the change in the net charge of the simulated system introduces large problems with long-range electrostatic corrections and the treatment of the solvent-exposed charged residues, as has been much discussed before and was also observed in the present simulations.^20,145^ Apparently, the consistent treatment of electronic relaxation and long-range electrostatic and dielectric relaxations of the surroundings in the QM + COSMO method provides a more balanced and much more effective treatment of redox processes than the current QM/MM or QTCP approaches.

Some previous studies employing grid-based or PB methods to treat the surrounding protein and solvent have reported lower errors of calculated redox potentials of iron–sulfur clusters in proteins, for example, root-mean-squared deviations of 0.03–0.14 and 0.01–0.04 V without and with MD simulations for six different types of iron–sulfur proteins with the protein-dipole Langevin-dipole approach, with or without MD simulations,^8^ 0.03–0.11 V in relative potentials for QM + PB calculations.^11,26,27,62,63,69−72^ The better performance is partly connected with the fact that only one type of iron–sulfur clusters are compared (if we translocate the results for each type of cluster individually, the MADtr value for our best method is reduced to 0.11 V; this is also illustrated by the τ_7_ measure, which is quite high for most methods). In future studies, we will also test these types of methods for our systems, also considering how sensitive the results are to the details and parameters of the calculations. We will also try to improve the present calculations, for example, by using different crystal structures for the reduced and oxidized states, because it has sometimes been observed that a change in oxidation state leads to conformational changes of some nearby protein groups.^36,41^

## Supporting Information Available

Setup of the studied proteins; calculated redox potentials for all methods; Fe–Fe, Fe–S, and Fe–N distances for all optimized structures; Mulliken spin populations of the various QM/MM structures; and computation times of the various methods (PDF)
Coordinates of the QM system for all the QM/MM optimized structures (ZIP)
