# Supplementary material for: Benchmark Study of Redox Potential Calculations for Iron–Sulfur Clusters in Proteins
Source: Inorg Chem. 2022 Apr 11;61(16):5991–6007. doi: 10.1021/acs.inorgchem.1c03422 (PMC9044450; doi:10.1021/acs.inorgchem.1c03422)
Supplement: Supplementary file 1 — ic1c03422_si_001.pdf [file ic1c03422_si_001.pdf]

Supporting Information

Benchmark Study of Redox Potential Calculations for Iron−Sulfur Clusters in Proteins

*Sonia Jafari,[1,2] Yakini A. Tavares Santos,[2] Justin Bergmann,[2] Mehdi Irani[1] and Ulf Ryde[2]**

[1] Department of Chemistry, University of Kurdistan, P.O. Box 66175-416, Sanandaj, Iran

[2] Department of Theoretical Chemistry, Lund University, Chemical Centre, P. O. Box 124, SE-221 00 Lund, Sweden

Correspondence to Ulf Ryde, E-mail: Ulf.Ryde@teokem.lu.se,

Tel: +46 – 46 2224502, Fax: +46 – 46 2228648

https://doi.org/10.1021/acs.inorgchem.1c03422

2022-04-03

Table S1. Setup of the studied proteins, including PDB structures used for the calculations, the abbreviation for each FeS system in the main article, the sizes of the QM and QM/MM systems (HL atoms are included in the atom counts of the QM systems), the protonation state of the His residues (HID, HIE and HIP refer that a His residue is protonated on ND1, NE2 and both on ND1 and NE2, respectively), and residues numbers of the Cys–Cys cross-links in the structures.

	PDB ID	1IRO	5NW3	1QT9	2PIA	2NUK	1FXD	5FD1	1IQZ	1FXR	5FD1	1CKU	2HIP
	Abb	Rub1	Rub2	2Fd1	2Fd2	Rieske	3Fd1	3Fd2	4Fd1	4Fd2	4Fd3	Hip1	Hip2
Number of atoms	QM/MM	7184	7151	7107	38996	12234	7057	12275	13415	12560	12275	13502	7053
QM	Min	33	33	36	36	44	31	31	40	40	40	40	40
	Int	114	117	133	132	154	151	138	156	146	149	139	139
	Big	212	218	243	293	288	268	284	306	293	323	341	364
The protonation states of His residues	HID	none	none	none	172<br>174	none	none	103	none	60	103	42	none
	HIE	none	none	none	171<br>195<br>214<br>221<br>291	85<br>106<br>109	none	35	none	none	35	none	8<br>22<br>50
	HIP	none	none	16<br>92	none	none	none	none	none	none	none	none	10
	Cys–Cys cross link	none	none	none	none	134-151	18-42	none	none	none	none	none	none

Table S2. Calculated redox potentials (V) from the QM/MM calculations.

Method	QM/MM; opt								QM/MM; sp																
Theory level	TPSS/def2-SV(P)								TPSS/def2-TZVPD						TPSS/aug-cc-pVTZ	B3LYP/def2-TZVPD		B3LYP/def2-SV(P)							
QM system	Min		Int				Big		Min		Int				Min	Min		Min		Int				Big	
Force filed	FF14SB		FF14SB		FF15IPQ		FF14SB		FF14SB		FF14SB		FF15IPQ		FF14SB	FF14SB		FF14SB		FF14SB		FF15IPQ		FF14SB	
System 2	Fix	Relax	Fix	Relax	Fix	Relax	Fix	Relax	Fix	Relax	Fix	Relax	Fix	Relax	Fix	Fix	Relax	Fix	Relax	Fix	Relax	Fix	Relax	Fix	Relax
Rub1	-12.9	-11.7	-11.0	-10.1	-9.4	-8.7	-9.5	-8.7	-12.2	-10.3	-10.5	-8.9	-9.0	-7.4	-12.1	-12.0	-10.0	-12.6	-10.5	-10.7	-9.1	-9.2	-7.6	-9.3	-8.2
Rub2	-8.8	-8.0	-11.0	-8.9	-9.8	-8.1	-9.6	-7.7	-8.2	-6.4	-10.4	-7.6	-9.2	-6.4	-8.1	-8.0	-6.3	-8.5	-6.7	-10.6	-7.7	-9.5	-6.6	-9.3	-6.3
2Fd1	-20.6	-18.7	-19.5	-18.1	-17.8	-15.5	-17.2	-15.3	-19.8	-19.3	-19.1	-18.4	-17.4	-15.3	-19.3	-19.8	-19.2	-20.3	-15.7	-19.1	-17.0	-17.4	-13.5	-16.8	-14.9
2Fd2	-7.2	-6.8	-5.1	-4.9	-3.9	-4.1	-3.2	-10.4	-6.5	-6.7	-5.0	-5.0	-3.7	-2.5	-6.3	-6.4	-6.6	-6.8	-4.9	-4.8	-3.8	-3.6	-2.6	-2.5	-1.7
Rieske	-2.7	-2.7	-3.1	-2.4	-2.4	-2.3	-3.7	-2.6	-2.9	-2.9	-2.7	-3.6	-2.4	-1.7	-2.6	-2.9	-2.7	-2.5	-2.0	-2.9	-1.7	-2.2	-1.1	-3.8	-2.7
3Fd1	-14.0	-13.6	-13.3	-12.6	-12.1	-11.4	-12.4	-12.3	-13.3	-13.4	-12.5	-12.0	-11.5	-10.2	-13.6	-12.5	-12.7	-12.9	-11.3	-12.2	-11.0	-11.0	-9.3	-11.3	-9.8
3Fd2	-16.9	-16.3	-15.5	-14.5	-13.7	-12.7	-14.0	-15.3	-16.1	-16.7	-13.4	-13.9	-13.5	-12.1	-16.0	-15.3	-15.1	-15.8	-14.1	-14.2	-12.8	-12.4	-11.0	-12.8	-10.9
4Fd1	-17.2	-15.9	-16.2	-14.9	-15.2	-13.8	-13.7	-13.2	-16.7	-16.0	-16.0	-15.2	-14.2	-13.5	-16.6	-16.5	-15.8	-16.8	-14.6	-15.9	-13.9	-15.6	-14.0	-13.4	-11.7
4Fd2	-14.8	-13.8	-14.0	-12.6	-12.2	-11.7	-11.8	-10.7	-14.2	-13.9	-18.3	-13.1	-11.2	-10.3	-14.2	-14.0	-13.8	-14.4	-12.5	-13.8	-11.0	-12.1	-9.7	-11.4	-9.4
4Fd3	-19.6	-18.7	-17.5	-16.8	-16.4	-15.9	-15.6	-15.2	-19.1	-18.6	-16.9	-16.5	-15.6	-15.0	-18.7	-18.9	-18.5	-19.2	-17.4	-17.9	-16.8	-16.5	-15.8	-15.2	-14.5
Hip1	-5.0	-4.7	-3.0	-3.4	-1.6	-0.8	-1.9	-2.4	-4.5	-4.5	-2.8	-2.0	-1.4	-0.7	-4.2	-4.4	-4.5	-5.6	-4.7	-2.1	-1.6	-1.4	-0.9	-0.9	0.1
Hip2	-9.4	-9.0	-7.9	-7.4	-7.1	-7.0	-7.8	-7.9	-9.1	-8.9	-7.4	-7.5	-6.7	-6.0	-8.6	-9.0	-8.8	-9.2	-7.9	-7.1	-6.5	-6.8	-6.2	-7.5	-7.1

Table S3. Calculated redox potentials (V) from the QTCP calculations.

Method	QTCP																		QTCP-noSurfaceCharge					
Theory level	TPSS/def2-SV(P)																		TPSS/def2-SV(P)					
QM system	Min						Intermediate												Min					
LR[a]	PB		GB		Ew		PB		GB		Ew		PB		GB		Ew		PB		GB		Ew	
SA[b]	Exc	Inc	Exc	Inc	Exc	Inc	Exc	Inc	Exc	Inc	Exc	Inc	Exc	Inc	Exc	Inc	Exc	Inc	Exc	Inc	Exc	Inc	Exc	Inc
Force filed	FF14SB						FF14SB						FF15IPQ						FF14SB					
Rub1	-7.94	-3.27	-4.62	-4.54	-5.00	-2.90	-6.16	-1.31	-3.13	-2.98	-3.25	-0.95	-4.75	0.34	-1.78	-1.62	-1.73	0.79	-5.74	-6.08	-4.37	-4.38	-4.89	-4.90
Rub2	-6.45	-4.15	-4.25	-4.07	-4.48	-3.38	-4.84	-0.07	-2.03	-1.84	-2.12	0.29	-4.00	1.38	-1.07	-0.82	-1.06	1.63	-5.34	-5.68	-4.17	-4.18	-4.48	-4.51
2Fd1	-9.03	-2.27	-4.10	-4.05	-4.32	-2.00	-7.49	0.27	-2.94	-2.61	-2.98	0.55	-3.81	4.38	0.91	1.16	0.95	4.37	-5.68	-5.36	-3.88	-3.87	-4.39	-4.10
2Fd2	-6.12	-3.92	-3.49	-3.52	-3.95	-3.30	-3.90	-2.26	-1.41	-1.48	-1.76	-1.62	-3.37	-1.79	-0.96	-1.05	-1.11	-1.03	-5.45	-4.21	-3.43	-3.31	-3.93	-3.21
Rieske	-3.16	-2.88	-1.94	-1.93	-1.60	-2.44	-4.18	-2.62	-3.33	-3.30	-1.23	-1.78	-2.87	-1.33	-2.34	-2.29	0.10	-0.45	-2.36	-1.70	-1.98	-1.96	-1.62	-1.32
3Fd1	-8.71	5.43	-2.80	-2.92	-3.15	1.40	-5.75	2.13	4.79	-1.54	-0.83	2.85	-4.89	2.91	0.45	0.39	0.15	3.71	-4.29	-4.32	-2.34	-2.34	-2.97	-2.98
3Fd2	-8.88	4.11	-2.46	-2.49	-3.34	2.94	-7.45	5.09	-1.60	-1.45	-1.99	3.91	-5.90	4.42	0.49	0.40	-0.28	4.25	-4.87	-0.16	-2.26	-1.79	-3.43	-0.27
4Fd1	-8.79	1.57	-3.57	-3.31	-3.96	1.62	-7.92	1.42	-3.12	-2.90	-3.26	1.62	-8.24	5.21	-2.02	-2.39	-2.37	2.04	-5.00	-5.30	-3.47	-3.48	-3.96	-3.99
4Fd2	-8.42	0.29	-3.62	-3.61	-4.23	0.46	3.02	10.93	7.69	7.25	7.20	11.39	1.29	9.12	6.24	5.41	5.59	9.70	-5.01	-5.33	-3.26	-3.27	-4.00	-4.04
4Fd3	-10.12	1.51	2.82	-6.74	-4.52	1.48	-7.68	0.55	-1.47	-1.33	-2.07	1.68	-4.17	4.65	2.32	2.07	1.58	5.62	-6.29	-4.68	-3.72	-3.97	-4.65	-3.95
Hip1	-4.62	-4.54	-2.52	-2.64	-3.22	-3.68	-2.20	-2.68	-0.89	-1.35	-0.80	-1.82	-0.36	-0.72	0.86	0.49	1.14	0.24	-4.08	-3.86	-2.18	-2.17	-3.06	-2.85
Hip2	-6.67	-2.02	0.48	-3.64	-3.63	-1.65	-4.55	-0.07	1.26	-2.41	-1.57	0.33	-3.24	2.45	-0.01	0.22	0.15	2.87	-4.70	-4.06	-2.68	-2.65	-3.36	-3.07

[a] LR is the long-range corrections, Poisson–Boltzmann, Generalised Born, or Ewald.

[b] SA is the treatment of solvent-accessible charged residues, excluded or included.

Table S4. Calculated redox potentials (V) from the QM+COSMO calculations (fixed surroundings). The TPSS/def2-TZVPD redox calculations were performed on structures optimized with TPSS/def2-SV(P).

Method	QM+COSMO																					
System 2	Fixed surroundings																					
Theory level	TPSS/def2-SV(P)													TPSS/def2-TZVPD						TPSS/aug-cc-pVTZ		
QM system	Min			Int						Big				Min			Int			Min		
Force filed	FF14SB			FF14SB			FF15IPQ			FF14SB				FF14SB			FF14SB			FF14SB		
ε	4	20	80	4	20	80	4	20	80	4	20	80	∞	4	20	80	4	20	80	4	20	80
Rub1	-3.4	-2.1	-1.8	-1.8	-1.0	-0.8	-1.8	-1.0	-0.8	-1.5	-0.8	-0.6	-0.6	-2.9	-1.6	-1.3	-1.5	-0.7	-0.5	-2.9	-1.6	-1.3
Rub2	-3.3	-2.0	-1.7	-1.7	-0.9	-0.8	-1.7	-0.9	-0.8	-1.4	-0.7	-0.5	-0.5	-2.6	-1.3	-1.0	-1.5	-0.7	-0.5	-2.8	-1.5	-1.2
2Fd1	-4.8	-2.7	-2.3	-3.0	-1.7	-1.5	-3.0	-1.7	-1.5	-2.6	-1.4	-1.2	-1.1	-5.4	-2.4	-1.7	-3.9	-0.9	-2.4	-4.1	-2.0	-1.6
2Fd2	-4.7	-2.7	-2.3	-2.4	-1.1	-0.8	-2.3	-1.1	-0.8	-3.1	-1.3	-0.8	-0.7	-4.1	-0.3	0.4	-0.5	0.7	1.0	-4.0	-2.0	-1.6
Rieske	-0.3	-0.2	-0.1	-0.4	-0.2	-0.2	-0.3	-0.2	-0.2	-0.6	-0.4	-0.3	-0.3	-1.5	-1.0	-0.9	1.5	1.7	1.7	-1.6	-1.0	-0.9
3Fd1	-3.4	-1.3	-0.9	-1.9	-0.6	-0.4	-1.9	-0.6	-0.4	-1.4	-0.6	-0.4	-0.3	-2.9	-0.9	-0.5	-3.2	-1.9	-1.7	-3.2	-1.2	-0.8
3Fd2	-3.4	-1.3	-0.9	-2.2	-0.8	-0.6	-2.2	-0.9	-0.6	-2.6	-1.1	-0.7	-0.6	-2.6	-0.6	-0.2	-1.2	0.1	0.4	-2.5	-0.5	-0.1
4Fd1	-4.0	-2.1	-1.7	-2.5	-1.3	-1.1	-2.9	-1.7	-1.4	-2.6	-1.5	-1.3	-1.2	-4.2	-1.9	-1.5	-2.2	-1.1	-0.9	-3.5	-1.7	-1.3
4Fd2	-4.0	-2.1	-1.7	-2.5	-1.3	-1.1	-2.5	-1.3	-1.1	-2.8	-1.7	-1.5	-1.4	-3.8	-1.9	-1.5	-3.5	-1.3	-0.7	-3.5	-1.6	-1.2
4Fd3	-4.1	-2.2	-1.8	-2.4	-1.6	-1.5	-2.4	-1.6	-1.4	-2.7	-1.5	-1.3	-1.2	-3.8	-1.9	-1.5	-1.3	-0.5	-0.3	-3.6	-1.7	-1.3
Hip1	-1.9	-0.7	-0.5	-0.8	-0.1	0.1	-0.8	0.0	0.1	-0.9	-0.3	-0.1	-0.1	-1.5	-2.0	-0.1	-0.7	0.1	0.3	-1.2	-0.1	0.2
Hip2	-1.7	-0.5	-0.2	-0.8	-0.1	0.1	-0.9	-0.1	0.1	-1.2	-0.6	-0.4	-0.4	-1.2	-0.1	0.2	0.3	-2.5	-2.3	-1.0	0.1	0.4

Table S4 Cont. Calculated redox potentials (V) from the QM+COSMO calculations (calculations with relaxed surroundings and redox potentials calculated with the same methods used for the geometry optimizations).

Method	QM+COSMO											
System 2	Relaxed surroundings											
Theory level	TPSS/def2-SV(P)											
QM system	Min			Int						Big		
Force filed	FF14SB			FF14SB			FF15IPQ			FF14SB		
ε	4	20	80	4	20	80	4	20	80	4	20	80
Rub1	-3.3	-2.0	-1.7	-2.0	-1.2	-1.1	-1.9	-1.1	-0.9	-1.4	-0.7	-0.5
Rub2	-3.3	-1.9	-1.7	-1.8	-0.9	-0.8	-1.8	-1.0	-0.8	-1.3	-0.5	-0.3
2Fd1	-5.1	-3.0	-2.6	-2.9	-1.7	-1.4	-3.2	-1.8	-1.5	-2.7	-1.5	-1.3
2Fd2	-4.7	-2.7	-2.2	-2.4	-1.1	-0.8	-2.4	-1.1	-0.8	-2.9	-1.0	-0.6
Rieske	-1.8	-1.2	-1.1	-0.2	-0.1	0.0	-0.5	-0.3	-0.3	-0.6	-0.2	-0.1
3Fd1	-3.2	-1.2	-0.7	-1.9	-0.6	-0.4	-1.9	-0.6	-0.3	-1.7	-0.9	-0.7
3Fd2	-3.3	-1.3	-0.8	-2.2	-0.9	-0.6	-2.2	-0.9	-0.6	-2.8	-1.2	-0.9
4Fd1	-4.1	-2.2	-1.8	-2.5	-1.3	-1.1	-2.9	-1.7	-1.5	-3.1	-1.9	-1.7
4Fd2	-4.1	-2.2	-1.8	-2.5	-1.2	-1.0	-2.7	-1.4	-1.2	-2.4	-1.3	-1.1
4Fd3	-4.1	-2.2	-1.8	-2.3	-1.6	-1.4	-2.3	-1.5	-1.4	-3.0	-1.9	-1.6
Hip1	-1.9	-0.7	-0.5	-0.8	0.0	0.1	-0.8	0.0	0.2	-1.0	-0.4	-0.2
Hip2	-1.6	-0.5	-0.2	-0.9	-0.1	0.0	-0.8	0.0	0.2	-1.2	-0.6	-0.4

Table S4. Cont. Calculated redox potentials (V) from the QM+COSMO calculations (redox potentials calculated with single-point calculations on geometries optimized at the TPSS/def2-SV(P) level of theory).

Method	QM+COSMO																							
Surroundings	Fixed surroundings												Relaxed surroundings											
Theory level	B3LYP/def2-SV(P)												B3LYP/def2-SV(P)											
QM system	Min			Int						Big			Min			Int						Big		
Force filed	FF14SB			FF14SB			FF15IPQ			FF14SB			FF14SB			FF14SB			FF15IPQ			FF14SB		
ε	4	20	80	4	20	80	4	20	80	4	20	80	4	20	80	4	20	80	4	20	80	4	20	80
Rub1	-3.1	-1.8	-1.5	-1.6	-0.8	-0.6	-1.6	-0.8	-0.6	-1.3	-0.6	-0.4	-3.1	-1.8	-1.5	-1.9	-1.1	-0.9	-1.7	-0.9	-0.8	-1.2	-0.5	-0.3
Rub2	-3.1	-1.8	-1.5	-1.5	-0.7	-0.6	-1.5	-0.7	-0.6	-1.2	-0.5	-0.3	-3.1	-1.7	-1.4	-1.6	-0.7	-0.6	-1.6	-0.8	-0.7	-1.0	-0.3	-0.1
2Fd1	-4.5	-2.5	-2.0	-2.8	-1.5	-1.2	-2.8	-1.5	-1.2	-2.3	-1.2	-0.9	-4.9	-2.8	-2.4	-2.7	-1.5	-1.3	-3.0	-1.5	-1.2	-2.4	-1.3	-1.0
2Fd2	-4.5	-2.4	-2.0	-2.1	-0.9	-0.6	-2.1	-0.8	-0.6	-3.1	-0.8	-0.4	-4.5	-2.4	-2.0	-2.2	-0.9	-0.6	-2.2	-0.9	-0.6	-2.7	-0.6	-0.3
Rieske	-1.6	-1.0	-0.9	-0.1	0.0	0.1	-0.1	0.0	0.1	-0.8	-0.6	-0.5	-1.6	-1.0	-0.9	0.0	0.2	0.2	-0.2	0.0	0.0	-0.4	-0.1	0.0
3Fd1	-2.3	-0.3	0.1	-0.9	0.3	0.6	-0.9	0.3	0.6	-0.4	0.4	0.6	-2.1	-0.1	0.3	-0.9	0.3	0.6	-0.9	0.3	0.6	-0.7	0.1	0.3
3Fd2	-2.3	-0.3	0.1	-1.1	0.3	0.6	-1.1	2.1	0.5	-1.5	0.0	0.4	-2.1	-0.1	0.3	-1.1	0.2	0.5	-1.1	0.2	0.5	-1.7	-0.2	0.1
4Fd1	-3.8	-1.9	-1.5	-2.3	-1.1	-0.9	-3.4	-2.2	-1.9	-2.4	-1.3	-1.1	-3.7	-1.9	-1.5	-2.3	-1.1	-0.8	-3.4	-2.2	-2.0	-2.8	-1.7	-1.4
4Fd2	-3.7	-1.8	-1.4	-2.3	-1.1	-0.9	-2.3	-1.1	-0.8	-2.4	-1.3	-1.1	-3.7	-1.8	-1.4	-2.3	-1.1	-0.8	-2.4	-1.2	-0.9	-1.9	-0.8	-0.6
4Fd3	-3.7	-1.8	-1.4	-2.9	-2.1	-1.9	-2.5	-1.7	-1.5	-2.3	-1.2	-1.0	-3.7	-1.8	-1.4	-2.8	-2.1	-1.9	-2.4	-1.6	-1.5	-2.7	-1.6	-1.4
Hip1	-2.4	-1.3	-1.0	0.0	0.7	0.9	-0.7	0.1	0.2	0.0	0.7	0.8	-2.4	-1.2	-1.0	0.0	0.8	0.9	-0.6	0.1	0.3	-0.1	0.5	0.6
Hip2	-1.5	-0.4	-0.1	-0.3	0.4	0.6	-0.7	0.0	0.2	-1.1	-0.4	-0.3	-1.5	-0.3	-0.1	-0.2	0.5	0.7	-0.6	0.1	0.3	-1.0	-0.4	-0.3

Table S5. Calculated Redox potentials (V) from QM+COSMO calculations, performed with the big QM system on structures optimized with the Min or Int QM system.

Method	QM+COSMO					
System 2	Fixed Surroundings					
Theory level	TPSS/def2-SV(P)					
QM system	Min			Int		
Force filed	FF14SB			FF14SB		
ε	4	20	80	4	20	80
Rub1	-2.2	-1.4	-1.2	-1.6	-0.8	-0.7
Rub2	-1.9	-1.1	-1.0	-1.6	-0.9	-0.7
2Fd1	-2.9	-1.7	-1.5	-2.9	-1.7	-1.4
2Fd2	-3.4	-1.5	-1.1	-3.3	-1.4	-0.9
Rieske	-0.5	-0.3	-0.2	-0.7	-0.4	-0.4
3Fd1	-1.2	-0.4	-0.2	-1.7	-0.9	-0.7
3Fd2	-2.7	-1.1	-0.8	-2.8	-1.1	-0.8
4Fd1	-2.8	-1.7	-1.4	-2.7	-1.6	-1.3
4Fd2	-3.1	-1.9	-1.7	-2.8	-1.7	-1.5
4Fd3	-3.2	-2.0	-1.7	-3.4	-2.2	-2.0
Hip1	-1.3	-0.6	-0.5	-1.1	-0.5	-0.3
Hip2	-1.0	-0.4	-0.2	-1.5	-0.8	-0.7

Table S6. Fe–Fe and Fe–S distances of the Min, Int, and Big systems in the optimized structures for Rub1. Atom names are defined in **Figure S1**.

QM system	1IRO, 1.1 Å	Min				Int								Big			
Force field		FF14SB				FF14SB				FF15IPQ				FF14SB			
Surroundings		Fix		Relax		Fix		Relax		Fix		Relax		Fix		Relax	
Oxidation state		Ox	Red	Ox	Red	Ox	Red	Ox	Red	Ox	Red	Ox	Red	Ox	Red	Ox	Red
Fe–SG(Cys-6)	2.29	2.28	2.33	2.29	2.37	2.29	2.34	2.29	2.36	2.30	2.35	2.31	2.35	2.32	2.37	2.31	2.34
Fe–SG(Cys-9)	2.25	2.25	2.27	2.26	2.30	2.27	2.28	2.28	2.28	2.26	2.28	2.26	2.27	2.27	2.30	2.27	2.29
Fe–SG(Cys-39)	2.28	2.30	2.34	2.31	2.38	2.34	2.38	2.33	2.35	2.33	2.37	2.32	2.35	2.30	2.33	2.29	2.31
Fe–SG(Cys-42)	2.23	2.24	2.27	2.26	2.30	2.25	2.28	2.26	2.29	2.24	2.27	2.25	2.28	2.26	2.29	2.26	2.29
	**MAD**	0.01	0.04	0.02	0.08	0.03	0.06	0.03	0.06	0.02	0.06	0.02	0.05	0.02	0.06	0.02	0.05

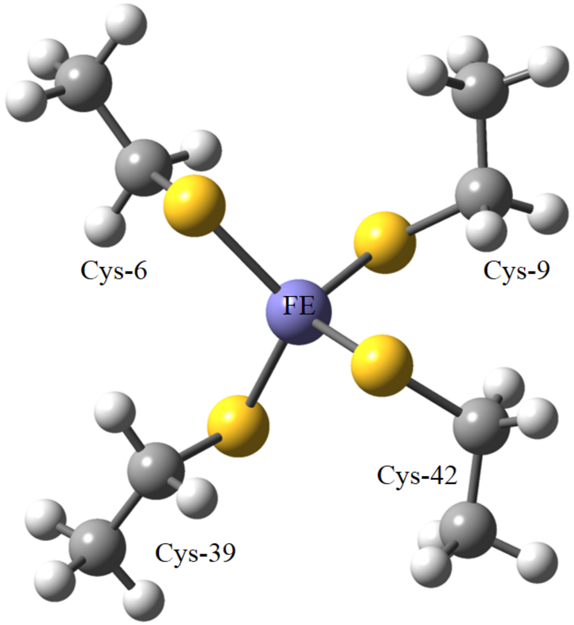

Figure S1. Min QM system for Rub1.

Table S7. Fe–Fe and Fe–S distances of the Min, Int, and Big systems in the optimized structures for Rub2. Atom names are defined in **Figure S2**.

QM system	5NW3, 0.59 Å	Min				Int								Big			
Force field		FF14SB				FF14SB				FF15IPQ				FF14SB			
Surroundings		Fix		Relax		Fix		Relax		Fix		Relax		Fix		Relax	
Oxidation state		Ox	Red	Ox	Red	Ox	Red	Ox	Red	Ox	Red	Ox	Red	Ox	Red	Ox	Red
Fe–SG(Cys-5)	2.30	2.29	2.32	2.30	2.34	2.30	2.36	2.30	2.34	2.31	2.36	2.30	2.33	2.32	2.36	2.30	2.33
Fe–SG(Cys-8)	2.27	2.26	2.28	2.26	2.30	2.26	2.28	2.27	2.28	2.26	2.28	2.26	2.27	2.27	2.30	2.28	2.30
Fe–SG(Cys-38)	2.32	2.30	2.33	2.31	2.34	2.32	2.36	2.33	2.36	2.32	2.36	2.32	2.34	2.30	2.33	2.30	2.32
Fe–SG(Cys-41)	2.28	2.28	2.32	2.29	2.33	2.25	2.29	2.26	2.30	2.25	2.28	2.25	2.26	2.24	2.28	2.24	2.29
	**MAD**	0.01	0.02	0.01	0.04	0.01	0.03	0.01	0.03	0.01	0.03	0.01	0.02	0.02	0.03	0.02	0.02

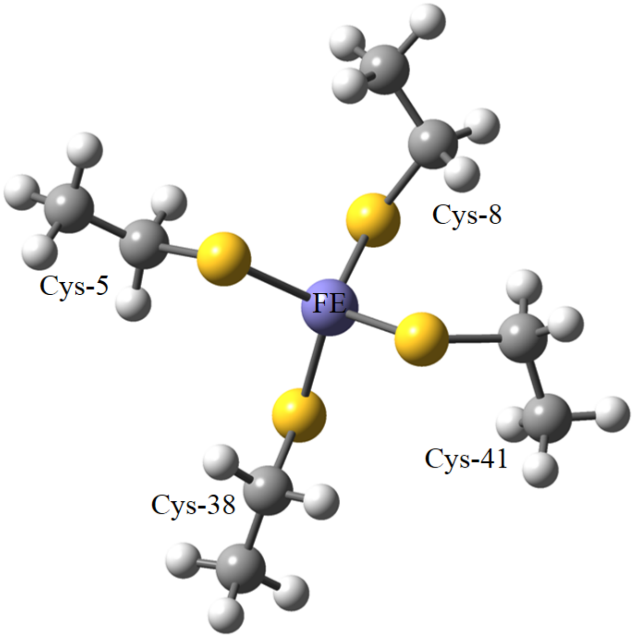

Figure S2. Min QM system for Rub2.

Table S8. Fe–Fe and Fe–S distances of the Min, Int, and Big systems in the optimized structures for 2Fd1. Atom names are defined in **Figure S3**.

QM system	1QT9, 1.3 Å	Min				Int								Big			
Force field		FF14SB				FF14SB				FF15IPQ				FF14SB			
Surroundings		Fix		Relax		Fix		Relax		Fix		Relax		Fix		Relax	
Oxidation state		Ox	Red	Ox	Red	Ox	Red	Ox	Red	Ox	Red	Ox	Red	Ox	Red	Ox	Red
Fe1–SG(Cys-41)	2.34	2.34	2.35	2.36	2.40	2.36	2.38	2.35	2.35	2.35	2.37	2.34	2.34	2.36	2.36	2.34	2.38
Fe1–SG(Cys-46)	2.28	2.29	2.30	2.29	2.34	2.28	2.28	2.27	2.30	2.28	2.28	2.28	2.28	2.29	2.29	2.29	2.30
Fe1–S1	2.28	2.25	2.32	2.26	2.29	2.26	2.34	2.24	2.30	2.27	2.35	2.25	2.29	2.25	2.30	2.25	2.29
Fe1–S2	2.24	2.17	2.25	2.18	2.22	2.17	2.22	2.17	2.22	2.17	2.21	2.17	2.18	2.16	2.21	2.16	2.20
Fe1–Fe2	2.74	2.67	2.69	2.71	2.64	2.68	2.70	2.63	2.64	2.67	2.69	2.65	2.62	2.66	2.68	2.64	2.66
Fe2–SG(Cys-49)	2.29	2.32	2.40	2.31	2.32	2.27	2.32	2.30	2.29	2.27	2.32	2.27	2.32	2.29	2.33	2.31	2.35
Fe2–SG(Cys-79)	2.29	2.26	2.30	2.25	2.29	2.26	2.29	2.27	2.30	2.25	2.28	2.27	2.27	2.26	2.29	2.27	2.29
Fe2–S1	2.22	2.27	2.28	2.27	2.25	2.25	2.26	2.24	2.25	2.25	2.27	2.24	2.26	2.25	2.26	2.25	2.26
Fe2–S2	2.19	2.20	2.19	2.20	2.20	2.17	2.18	2.20	2.19	2.17	2.18	2.18	2.19	2.16	2.17	2.17	2.18
	**MAD**	0.03	0.03	0.03	0.03	0.03	0.03	0.03	0.02	0.03	0.03	0.03	0.03	0.03	0.03	0.03	0.03

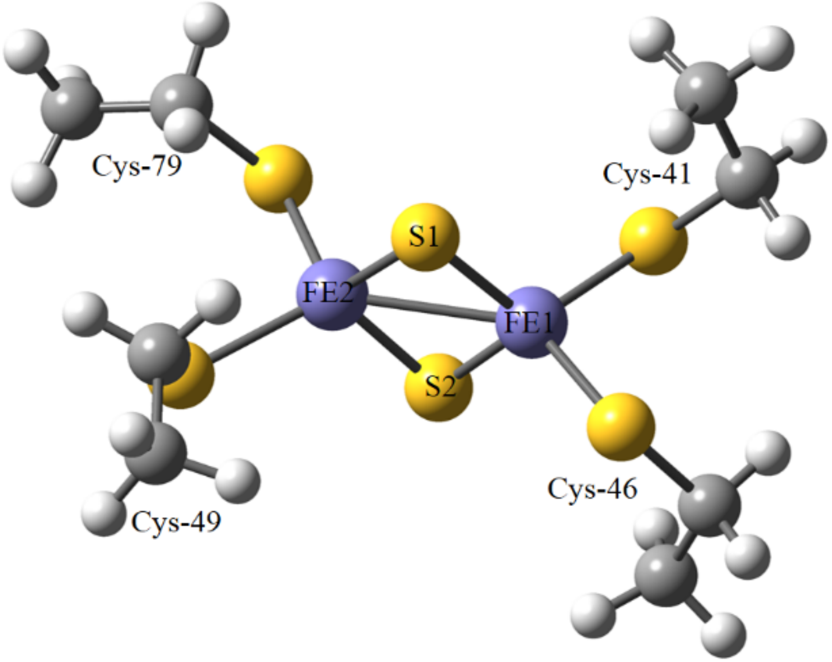

Figure S3. Min QM system for 2Fd1.

Table S9. Fe–Fe and Fe–S distances of the Min, Int, and Big systems in the optimized structures for 2Fd2. Atom names are defined in **Figure S4**.

QM system	2PIA, 2.0 Å	Min				Int								Big			
Force field		FF14SB				FF14SB				FF15IPQ				FF14SB			
Surroundings		Fix		Relax		Fix		Relax		Fix		Relax		Fix		Relax	
Oxidation state		Ox	Red	Ox	Red	Ox	Red	Ox	Red	Ox	Red	Ox	Red	Ox	Red	Ox	Red
Fe1–SG(Cys-272)	2.32	2.35	2.40	2.35	2.37	2.33	2.39	2.33	2.38	2.33	2.39	2.33	2.38	2.36	2.40	2.36	2.40
Fe1–SG(Cys-277)	2.35	2.32	2.34	2.33	2.34	2.33	2.32	2.33	2.29	2.32	2.31	2.29	2.30	2.33	2.32	2.30	2.31
Fe1–S1	2.15	2.22	2.28	2.21	2.28	2.21	2.25	2.23	2.26	2.22	2.26	2.24	2.27	2.24	2.26	2.24	2.27
Fe1–S2	2.08	2.21	2.27	2.21	2.25	2.19	2.22	2.19	2.23	2.19	2.22	2.20	2.22	2.21	2.23	2.20	2.22
Fe1–Fe2	2.64	2.66	2.62	2.68	2.68	2.65	2.61	2.62	2.59	2.64	2.61	2.60	2.55	2.64	2.62	2.63	2.61
Fe2–SG(Cys-280)	2.33	2.30	2.38	2.28	2.35	2.28	2.34	2.25	2.29	2.28	2.34	2.25	2.29	2.30	2.33	2.28	2.32
Fe2–SG(Cys-308)	2.16	2.29	2.33	2.31	2.37	2.28	2.31	2.28	2.32	2.28	2.31	2.28	2.31	2.30	2.32	2.32	2.33
Fe2–S1	2.17	2.26	2.28	2.25	2.25	2.24	2.26	2.23	2.25	2.24	2.26	2.23	2.26	2.24	2.25	2.24	2.25
Fe2–S2	2.19	2.25	2.26	2.25	2.26	2.23	2.23	2.22	2.24	2.22	2.23	2.22	2.23	2.21	2.22	2.22	2.22
	**MAD**	0.07	0.09	0.07	0.09	0.05	0.07	0.06	0.08	0.05	0.08	0.07	0.09	0.06	0.07	0.07	0.08

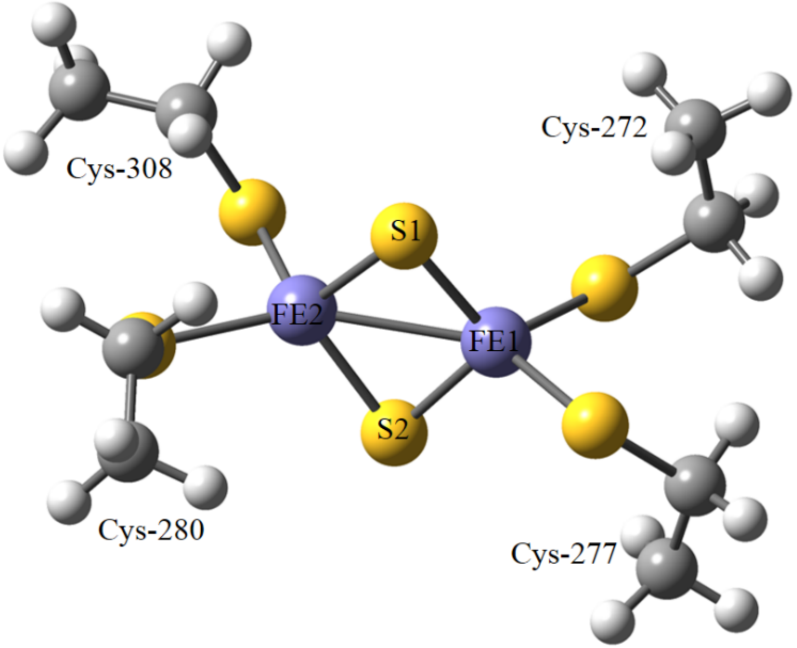

Figure S4. Min QM system for 2Fd2.

Table S10. Fe–Fe, Fe–S, and Fe-N distances of the Min, Int, and Big systems in the optimized structures for the Rieske protein. Atom names are defined in **Figure S5**.

QM system	2NUK, 1.2 Å	Min				Int								Big			
Force field		FF14SB				FF14SB				FF15IPQ				FF14SB			
Surroundings		Fix		Relax		Fix		Relax		Fix		Relax		Fix		Relax	
Oxidation state		Ox	Red	Ox	Red	Ox	Red	Ox	Red	Ox	Red	Ox	Red	Ox	Red	Ox	Red
Fe1–SG(Cys-129)	2.33	2.25	2.29	2.26	2.34	2.29	2.32	2.28	2.32	2.30	2.33	2.29	2.32	2.31	2.33	2.27	2.28
Fe1–SG(Cys-149)	2.29	2.22	2.27	2.24	2.29	2.25	2.28	2.26	2.29	2.25	2.28	2.26	2.29	2.27	2.30	2.26	2.28
Fe1–S1	2.20	2.22	2.23	2.22	2.24	2.20	2.21	2.18	2.20	2.20	2.20	2.18	2.19	2.21	2.22	2.19	2.21
Fe1–S2	2.20	2.18	2.20	2.21	2.23	2.19	2.20	2.20	2.21	2.19	2.20	2.21	2.20	2.20	2.21	2.18	2.20
Fe1–Fe2	2.69	2.59	2.53	2.68	2.64	2.61	2.55	2.66	2.60	2.60	2.54	2.67	2.63	2.64	2.59	2.57	2.53
Fe2-ND1(HIE-131)	2.12	2.02	2.05	2.06	2.12	2.04	2.07	2.06	2.10	2.03	2.07	2.07	2.10	2.06	2.10	2.02	2.06
Fe2-ND1(HIE-152)	2.10	2.01	2.04	2.04	2.09	2.00	2.04	2.05	2.09	2.00	2.04	2.03	2.10	2.02	2.07	2.02	2.05
Fe2–S1	2.21	2.18	2.20	2.20	2.23	2.19	2.21	2.19	2.23	2.18	2.21	2.19	2.23	2.22	2.25	2.22	2.25
Fe2–S2	2.22	2.18	2.22	2.21	2.24	2.19	2.23	2.23	2.27	2.19	2.23	2.23	2.28	2.20	2.25	2.20	2.23
	**MAD**	0.061	0.04	0.03	0.02	0.04	0.03	0.03	0.02	0.05	0.03	0.03	0.02	0.03	0.03	0.05	0.04

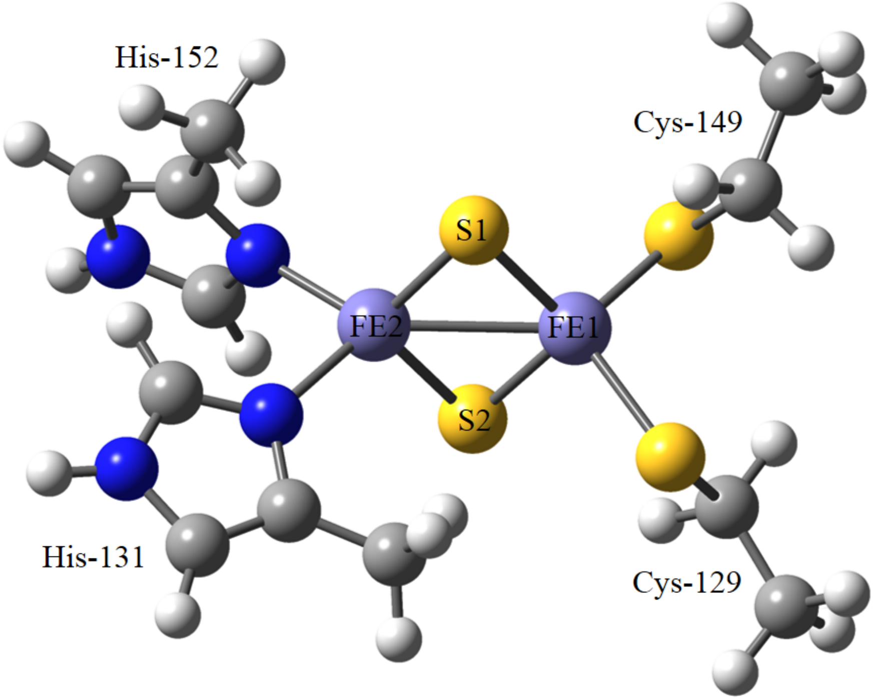

Figure S5. Min QM system for Rieske protein.

Table S11. Fe–Fe and Fe–S distances of the Min, Int, and Big systems in the optimized structures for 3Fd1. Atom names are defined in **Figure S6**.

QM system	1FXD, 1.7 Å	Min				Int								Big			
Force field		FF14SB				FF14SB				FF15IPQ				FF14SB			
Surroundings		Fix		Relax		Fix		Relax		Fix		Relax		Fix		Relax	
Oxidation state		Ox	Red	Ox	Red	Ox	Red	Ox	Red	Ox	Red	Ox	Red	Ox	Red	Ox	Red
Fe1–SG(Cys-8))	2.28	2.22	2.30	2.27	2.31	2.23	2.30	2.23	2.31	2.24	2.31	2.22	2.32	2.23	2.30	2.21	2.30
Fe1–S1	2.23	2.18	2.23	2.22	2.23	2.18	2.20	2.17	2.21	2.18	2.20	2.16	2.21	2.17	2.22	2.16	2.22
Fe1–S2	2.32	2.21	2.27	2.24	2.25	2.24	2.30	2.25	2.31	2.24	2.30	2.24	2.31	2.22	2.28	2.21	2.29
Fe1–S3	2.32	2.23	2.25	2.30	2.25	2.23	2.23	2.22	2.22	2.23	2.22	2.21	2.22	2.22	2.22	2.20	2.22
Fe1–Fe3	2.70	2.65	2.64	2.65	2.67	2.66	2.65	2.66	2.66	2.66	2.65	2.66	2.65	2.66	2.65	2.67	2.67
Fe1–Fe4	2.75	2.64	2.66	2.64	2.66	2.63	2.63	2.63	2.62	2.63	2.63	2.63	2.63	2.64	2.64	2.64	2.64
Fe3–SG(Cys-14))	2.28	2.18	2.29	2.23	2.33	2.18	2.27	2.20	2.29	2.18	2.27	2.20	2.28	2.20	2.30	2.23	2.32
Fe3–S1	2.26	2.17	2.24	2.20	2.25	2.17	2.25	2.17	2.24	2.17	2.25	2.18	2.24	2.17	2.25	2.18	2.24
Fe3–S3	2.32	2.23	2.35	2.28	2.36	2.23	2.35	2.25	2.36	2.23	2.35	2.26	2.36	2.23	2.34	2.26	2.35
Fe3–S4	2.27	2.21	2.32	2.20	2.31	2.21	2.33	2.21	2.33	2.21	2.33	2.21	2.32	2.21	2.33	2.22	2.34
Fe3–Fe4	2.78	2.64	2.60	2.62	2.61	2.64	2.60	2.64	2.61	2.64	2.59	2.64	2.60	2.64	2.59	2.64	2.59
Fe4–SG(Cys-50)	2.22	2.28	2.38	2.21	2.38	2.26	2.34	2.26	2.35	2.25	2.33	2.27	2.36	2.25	2.33	2.27	2.34
Fe4–S2	2.29	2.25	2.27	2.25	2.28	2.25	2.26	2.26	2.26	2.25	2.26	2.25	2.26	2.24	2.25	2.26	2.25
Fe4–S3	2.31	2.31	2.35	2.24	2.36	2.31	2.32	2.30	2.31	2.31	2.32	2.29	2.31	2.31	2.32	2.31	2.32
Fe4–S4	2.22	2.29	2.34	2.19	2.34	2.23	2.29	2.23	2.28	2.23	2.29	2.23	2.28	2.24	2.30	2.23	2.30
	**MAD**	0.07	0.06	0.05	0.06	0.07	0.06	0.06	0.06	0.07	0.06	0.07	0.06	0.07	0.06	0.07	0.06

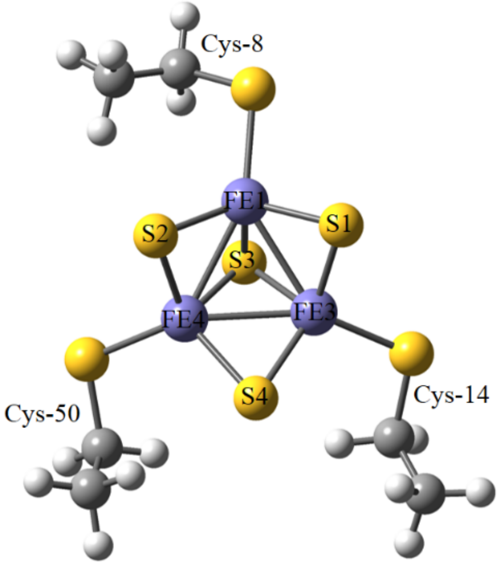

Figure S6. Min QM system for 3Fd1.

Table S12. Fe–Fe and Fe–S distances of the Min, Int, and Big systems in the optimized structures for 3Fd2. Atom names are defined in **Figure S7**.

QM system	5FD1, 1.9 Å	Min				Int								Big			
Force field		FF14SB				FF14SB				FF15IPQ				FF14SB			
Surroundings		Fix		Relax		Fix		Relax		Fix		Relax		Fix		Relax	
Oxidation state		Ox	Red	Ox	Red	Ox	Red	Ox	Red	Ox	Red	Ox	Red	Ox	Red	Ox	Red
Fe1–SG(Cys-16)	2.31	2.24	2.30	2.24	2.34	2.21	2.26	2.23	2.28	2.21	2.26	2.23	2.28	2.22	2.27	2.24	2.32
Fe1–S1	2.30	2.25	2.27	2.23	2.28	2.24	2.25	2.23	2.24	2.24	2.25	2.23	2.25	2.23	2.24	2.24	2.24
Fe1–S2	2.25	2.25	2.31	2.21	2.32	2.25	2.31	2.26	2.31	2.25	2.32	2.26	2.31	2.27	2.34	2.27	2.34
Fe1–S3	2.29	2.31	2.35	2.27	2.37	2.31	2.34	2.30	2.33	2.31	2.33	2.30	2.33	2.31	2.34	2.31	2.35
Fe1–Fe3	2.71	2.64	2.66	2.66	2.67	2.64	2.65	2.65	2.65	2.64	2.64	2.65	2.65	2.65	2.65	2.67	2.69
Fe1–Fe4	2.64	2.63	2.57	2.62	2.59	2.64	2.59	2.65	2.60	2.64	2.60	2.65	2.61	2.64	2.59	2.66	2.60
Fe3–SG(Cys-8)	2.27	2.20	2.29	2.19	2.30	2.23	2.29	2.23	2.30	2.23	2.29	2.24	2.29	2.20	2.26	2.20	2.26
Fe3–S1	2.31	2.19	2.24	2.14	2.22	2.17	2.21	2.19	2.23	2.18	2.21	2.20	2.23	2.18	2.22	2.17	2.21
Fe3–S3	2.27	2.26	2.27	2.22	2.27	2.27	2.25	2.28	2.25	2.27	2.24	2.27	2.24	2.27	2.25	2.25	2.25
Fe3–S4	2.30	2.18	2.24	2.17	2.25	2.20	2.26	2.22	2.27	2.19	2.26	2.22	2.28	2.20	2.27	2.18	2.27
Fe3–Fe4	2.64	2.64	2.65	2.64	2.65	2.65	2.65	2.64	2.63	2.65	2.65	2.64	2.64	2.65	2.64	2.64	2.62
Fe4–SG(Cys-49)	2.29	2.22	2.39	2.29	2.40	2.24	2.38	2.25	2.39	2.24	2.38	2.23	2.38	2.23	2.36	2.22	2.35
Fe4–S2	2.26	2.24	2.35	2.25	2.32	2.19	2.31	2.19	2.31	2.19	2.32	2.19	2.31	2.18	2.30	2.19	2.30
Fe4–S3	2.27	2.23	2.36	2.32	2.35	2.21	2.33	2.20	2.32	2.20	2.33	2.19	2.31	2.21	2.32	2.22	2.33
Fe4–S4	2.28	2.18	2.26	2.26	2.27	2.18	2.26	2.17	2.25	2.18	2.26	2.17	2.25	2.18	2.23	2.18	2.22
	**MAD**	0.05	0.06	0.05	0.06	0.05	0.05	0.05	0.05	0.06	0.05	0.05	0.05	0.06	0.05	0.06	0.05

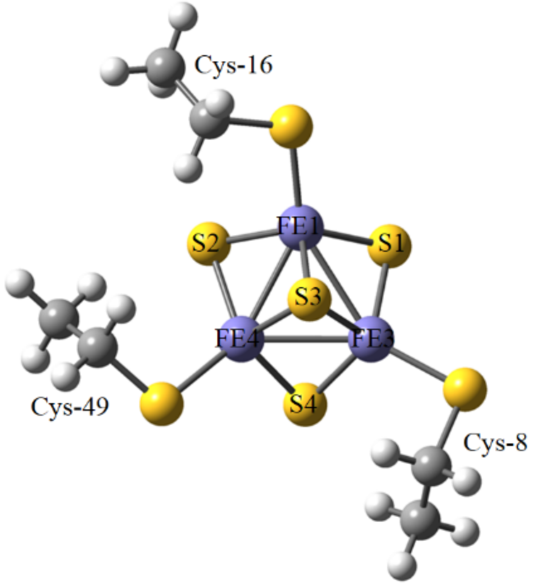

Figure S7. Min QM system for 3Fd2.

Table S13. Fe–Fe and Fe–S distances of the Min, Int, and Big systems in the optimized structures for 4Fd1. Atom names are defined in **Figure S8**.

QM system	1IQZ, 0.92 Å	Min				Int								Big			
Force field		FF14SB				FF14SB				FF15IPQ				FF14SB			
Surroundings		Fix		Relax		Fix		Relax		Fix		Relax		Fix		Relax	
Oxidation state		Ox	Red	Ox	Red	Ox	Red	Ox	Red	Ox	Red	Ox	Red	Ox	Red	Ox	Red
Fe1–SG(Cys-61)	2.27	2.29	2.32	2.32	2.37	2.28	2.32	2.28	2.32	2.28	2.31	2.28	2.31	2.28	2.30	2.28	2.30
Fe1–S2	2.28	2.25	2.30	2.26	2.29	2.22	2.25	2.22	2.24	2.24	2.24	2.24	2.23	2.22	2.25	2.21	2.23
Fe1–S3	2.30	2.34	2.35	2.34	2.35	2.32	2.32	2.33	2.32	2.36	2.32	2.36	2.32	2.32	2.32	2.32	2.31
Fe1–S4	2.29	2.34	2.35	2.32	2.35	2.33	2.34	2.32	2.33	2.36	2.33	2.36	2.32	2.31	2.33	2.30	2.31
Fe1–Fe4	2.72	2.63	2.65	2.66	2.67	2.59	2.60	2.58	2.59	2.78	2.60	2.76	2.58	2.59	2.60	2.58	2.59
Fe1–Fe2	2.71	2.63	2.63	2.71	2.73	2.62	2.61	2.61	2.61	2.77	2.61	2.78	2.60	2.65	2.63	2.63	2.64
Fe1–Fe3	2.73	2.64	2.65	2.67	2.69	2.62	2.63	2.63	2.64	2.78	2.63	2.78	2.64	2.61	2.62	2.62	2.63
Fe3–SG(Cys-17)	2.27	2.25	2.31	2.28	2.33	2.24	2.29	2.25	2.29	2.23	2.27	2.24	2.27	2.24	2.28	2.24	2.28
Fe3–S2	2.31	2.33	2.36	2.33	2.36	2.32	2.33	2.32	2.34	2.35	2.34	2.35	2.34	2.32	2.35	2.33	2.35
Fe3–S4	2.27	2.25	2.26	2.25	2.27	2.26	2.27	2.25	2.27	2.28	2.26	2.27	2.26	2.24	2.25	2.24	2.26
Fe3–S1	2.31	2.36	2.39	2.36	2.38	2.33	2.36	2.34	2.37	2.35	2.36	2.35	2.37	2.34	2.36	2.34	2.36
Fe3–Fe2	2.74	2.64	2.61	2.64	2.61	2.66	2.62	2.67	2.62	2.79	2.64	2.78	2.65	2.68	2.65	2.66	2.65
Fe3–Fe4	2.72	2.62	2.56	2.69	2.63	2.63	2.58	2.64	2.60	2.78	2.58	2.80	2.63	2.63	2.57	2.65	2.58
Fe2–SG(Cys-14)	2.27	2.27	2.27	2.28	2.29	2.30	2.29	2.30	2.28	2.29	2.29	2.29	2.29	2.30	2.29	2.28	2.28
Fe2–S3	2.29	2.31	2.32	2.31	2.33	2.30	2.32	2.29	2.31	2.33	2.32	2.32	2.30	2.30	2.31	2.29	2.31
Fe2–S4	2.31	2.35	2.37	2.36	2.39	2.35	2.38	2.36	2.39	2.37	2.36	2.37	2.37	2.35	2.36	2.35	2.38
Fe2–S1	2.28	2.26	2.31	2.26	2.30	2.25	2.29	2.24	2.28	2.27	2.29	2.26	2.28	2.24	2.29	2.23	2.27
Fe2–Fe4	2.73	2.66	2.66	2.66	2.66	2.63	2.63	2.63	2.62	2.78	2.63	2.78	2.63	2.63	2.64	2.61	2.62
Fe4–SG(Cys-11)	2.30	2.30	2.36	2.30	2.37	2.29	2.34	2.28	2.34	2.29	2.34	2.29	2.32	2.29	2.33	2.28	2.32
Fe4–S2	2.28	2.31	2.33	2.31	2.32	2.29	2.30	2.28	2.29	2.32	2.30	2.32	2.29	2.29	2.30	2.28	2.29
Fe4–S3	2.27	2.24	2.25	2.24	2.26	2.24	2.25	2.24	2.26	2.28	2.26	2.28	2.26	2.24	2.26	2.24	2.26
Fe4–S1	2.29	2.33	2.34	2.33	2.34	2.32	2.34	2.32	2.34	2.35	2.33	2.34	2.32	2.32	2.33	2.31	2.33
	**MAD**	0.04	0.06	0.03	0.05	0.04	0.05	0.05	0.05	0.04	0.05	0.04	0.05	0.04	0.05	0.04	0.05

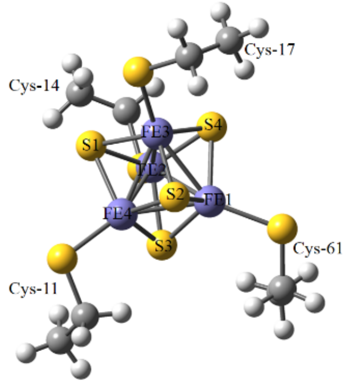

Figure S8. Min QM system for 4Fd1.

Table S14. Fe–Fe and Fe–S distances of the Min, Int, and Big systems in the optimized structures for 4Fd2. Atom names are defined in **Figure S9**.

QM system	1FXR, 2.3 Å	Min				Int								Big			
Force field		FF14SB				FF14SB				FF15IPQ				FF14SB			
Surroundings		Fix		Relax		Fix		Relax		Fix		Relax		Fix		Relax	
Oxidation state		Ox	Red	Ox	Red	Ox	Red	Ox	Red	Ox	Red	Ox	Red	Ox	Red	Ox	Red
Fe1–SG(Cys-11)	2.31	2.30	2.36	2.30	2.36	2.31	2.35	2.33	2.37	2.31	2.34	2.32	2.35	2.27	2.30	2.30	2.30
Fe1–S2	2.31	2.27	2.29	2.26	2.27	2.26	2.27	2.25	2.26	2.29	2.30	2.28	2.28	2.38	2.38	2.36	2.37
Fe1–S3	2.28	2.34	2.36	2.34	2.36	2.29	2.30	2.30	2.30	2.32	2.32	2.32	2.32	2.32	2.33	2.33	2.34
Fe1–S4	2.32	2.36	2.36	2.37	2.38	2.32	2.33	2.33	2.33	2.35	2.36	2.34	2.35	2.26	2.33	2.27	2.33
Fe1–Fe3	2.80	2.80	2.79	2.82	2.80	2.64	2.63	2.67	2.65	2.80	2.81	2.83	2.83	2.80	2.81	2.81	2.82
Fe1–Fe2	2.73	2.77	2.74	2.77	2.74	2.64	2.60	2.70	2.63	2.78	2.76	2.78	2.77	2.75	2.75	2.78	2.77
Fe1–Fe4	2.70	2.75	2.75	2.74	2.74	2.59	2.61	2.60	2.61	2.75	2.76	2.74	2.74	2.80	2.76	2.77	2.74
Fe2–SG(Cys-17)	2.23	2.26	2.32	2.27	2.32	2.23	2.27	2.24	2.27	2.23	2.25	2.23	2.26	2.25	2.29	2.26	2.31
Fe2–S1	2.30	2.25	2.25	2.25	2.25	2.22	2.23	2.22	2.24	2.25	2.26	2.25	2.27	2.32	2.34	2.33	2.35
Fe2–S3	2.30	2.35	2.37	2.34	2.37	2.32	2.33	2.31	2.32	2.35	2.35	2.33	2.33	2.26	2.28	2.25	2.29
Fe2–S4	2.34	2.38	2.40	2.37	2.39	2.33	2.36	2.33	2.36	2.35	2.37	2.35	2.36	2.37	2.39	2.35	2.39
Fe2–Fe3	2.76	2.76	2.76	2.76	2.76	2.62	2.60	2.61	2.59	2.77	2.77	2.76	2.76	2.81	2.77	2.76	2.74
Fe2–Fe4	2.75	2.79	2.79	2.80	2.80	2.65	2.66	2.66	2.67	2.80	2.80	2.81	2.82	2.79	2.79	2.81	2.80
Fe3–SG(Cys-14)	2.00	2.24	2.23	2.23	2.21	2.25	2.24	2.26	2.26	2.25	2.24	2.26	2.26	2.26	2.30	2.26	2.30
Fe3–S1	2.31	2.36	2.40	2.35	2.40	2.33	2.34	2.33	2.35	2.35	2.36	2.35	2.37	2.34	2.35	2.36	2.36
Fe3–S2	2.30	2.34	2.33	2.35	2.35	2.30	2.30	2.30	2.32	2.34	2.33	2.34	2.34	2.25	2.27	2.26	2.27
Fe3–S4	2.27	2.28	2.34	2.28	2.33	2.25	2.28	2.26	2.28	2.27	2.31	2.28	2.32	2.34	2.35	2.36	2.36
Fe3–Fe4	2.66	2.76	2.75	2.75	2.73	2.61	2.60	2.56	2.57	2.78	2.76	2.76	2.74	2.76	2.76	2.79	2.78
Fe4–SG(Cys-54)	2.14	2.30	2.34	2.30	2.34	2.29	2.32	2.30	2.34	2.28	2.31	2.30	2.33	2.27	2.30	2.28	2.28
Fe4–S1	2.33	2.36	2.38	2.36	2.36	2.31	2.31	2.31	2.30	2.34	2.35	2.35	2.35	2.26	2.32	2.26	2.30
Fe4–S2	2.32	2.37	2.37	2.38	2.39	2.34	2.33	2.35	2.35	2.36	2.36	2.37	2.38	2.38	2.42	2.40	2.42
Fe4–S3	2.31	2.29	2.34	2.31	2.35	2.24	2.28	2.25	2.28	2.27	2.29	2.28	2.29	2.35	2.34	2.38	2.35
	**MAD**	0.05	0.06	0.05	0.06	0.06	0.07	0.06	0.07	0.05	0.05	0.05	0.06	0.06	0.06	0.06	0.06

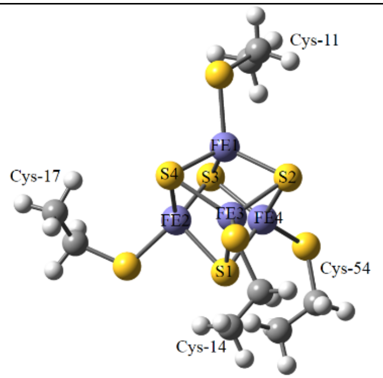

Figure S9. Min QM system for 4Fd2.

Table S15. Fe–Fe and Fe–S distances of the Min, Int, and Big systems in the optimized structures for 4Fd3. Atom names are defined in **Figure S10**.

QM system	5FD1, 1.9 Å	Min				Int								Big			
Force field		FF14SB				FF14SB				FF15IPQ				FF14SB			
Surroundings		Fix		Relax		Fix		Relax		Fix		Relax		Fix		Relax	
Oxidation state		Ox	Red	Ox	Red	Ox	Red	Ox	Red	Ox	Red	Ox	Red	Ox	Red	Ox	Red
Fe1–SG(Cys-39)	2.31	2.31	2.32	2.33	2.32	2.28	2.28	2.29	2.27	2.28	2.27	2.28	2.27	2.28	2.26	2.28	2.26
Fe1–S2	2.31	2.32	2.33	2.32	2.33	2.31	2.29	2.30	2.29	2.31	2.33	2.30	2.31	2.30	2.30	2.30	2.30
Fe1–S3	2.32	2.26	2.31	2.25	2.30	2.27	2.28	2.28	2.30	2.28	2.33	2.28	2.33	2.29	2.35	2.29	2.37
Fe1–S4	2.30	2.34	2.34	2.35	2.37	2.34	2.31	2.33	2.30	2.34	2.35	2.32	2.34	2.34	2.34	2.33	2.33
Fe1–Fe2	2.75	2.80	2.82	2.80	2.80	2.80	2.65	2.80	2.64	2.80	2.81	2.80	2.80	2.80	2.81	2.80	2.81
Fe1–Fe3	2.64	2.78	2.75	2.80	2.78	2.80	2.62	2.82	2.65	2.80	2.77	2.83	2.83	2.81	2.78	2.83	2.80
Fe1–Fe4	2.75	2.76	2.76	2.74	2.75	2.75	2.60	2.73	2.57	2.75	2.74	2.73	2.70	2.75	2.76	2.75	2.75
Fe2–SG(Cys-42)	2.30	2.28	2.34	2.29	2.35	2.30	2.35	2.31	2.36	2.30	2.34	2.31	2.36	2.32	2.35	2.31	2.35
Fe2–S1	2.25	2.33	2.35	2.33	2.35	2.33	2.33	2.33	2.33	2.33	2.33	2.33	2.33	2.32	2.33	2.33	2.33
Fe2–S3	2.27	2.32	2.34	2.34	2.35	2.32	2.30	2.33	2.31	2.32	2.32	2.33	2.32	2.32	2.33	2.33	2.34
Fe2–S4	2.31	2.28	2.29	2.27	2.28	2.27	2.26	2.28	2.27	2.27	2.27	2.28	2.28	2.26	2.27	2.26	2.27
Fe2–Fe3	2.70	2.76	2.77	2.75	2.76	2.77	2.62	2.75	2.63	2.77	2.63	2.75	2.65	2.76	2.78	2.74	2.76
Fe2–Fe4	2.72	2.77	2.73	2.78	2.73	2.78	2.57	2.79	2.57	2.78	2.59	2.80	2.59	2.80	2.76	2.82	2.77
Fe3–SG(Cys-45)	2.29	2.23	2.25	2.26	2.29	2.21	2.24	2.23	2.25	2.21	2.23	2.23	2.24	2.22	2.24	2.23	2.25
Fe3–S1	2.28	2.25	2.31	2.24	2.28	2.25	2.27	2.24	2.26	2.25	2.27	2.24	2.25	2.24	2.29	2.23	2.27
Fe3–S2	2.27	2.35	2.35	2.33	2.33	2.34	2.30	2.32	2.29	2.34	2.31	2.32	2.30	2.33	2.34	2.32	2.32
Fe3–S4	2.29	2.37	2.40	2.36	2.39	2.34	2.33	2.34	2.33	2.34	2.33	2.33	2.34	2.33	2.35	2.33	2.35
Fe3–Fe4	2.72	2.79	2.79	2.78	2.77	2.79	2.63	2.79	2.64	2.79	2.63	2.79	2.65	2.80	2.80	2.80	2.80
Fe4–SG(Cys-20)	2.31	2.31	2.38	2.33	2.42	2.30	2.37	2.31	2.37	2.30	2.36	2.31	2.36	2.30	2.35	2.31	2.36
Fe4–S1	2.28	2.37	2.39	2.36	2.37	2.37	2.35	2.38	2.36	2.37	2.35	2.38	2.36	2.37	2.39	2.37	2.39
Fe4–S2	2.31	2.27	2.29	2.29	2.31	2.25	2.23	2.25	2.24	2.25	2.24	2.25	2.24	2.25	2.26	2.26	2.26
Fe4–S3	2.29	2.35	2.36	2.36	2.38	2.35	2.35	2.36	2.36	2.35	2.36	2.37	2.37	2.35	2.37	2.36	2.39
	**MAD**	0.05	0.05	0.05	0.05	0.05	0.06	0.05	0.06	0.05	0.06	0.05	0.06	0.05	0.06	0.05	0.06

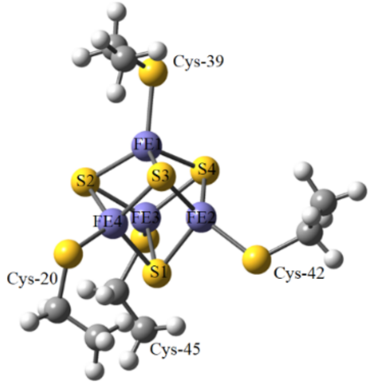

Figure S10. Min QM system for 4Fd3.

Table S16. Fe–Fe and Fe–S distances of the Min, Int, and Big systems in the optimized structures for Hip1. Atom names are defined in **Figure S11**.

QM system	1CKU, 1.2 Å	Min				Int								Big			
Force field		FF14SB				FF14SB				FF15IPQ				FF14SB			
Surroundings		Fix		Relax		Fix		Relax		Fix		Relax		Fix		Relax	
Oxidation state		Ox	Red	Ox	Red	Ox	Red	Ox	Red	Ox	Red	Ox	Red	Ox	Red	Ox	Red
Fe1–SG(Cys-43)	2.25	2.21	2.26	2.20	2.25	2.25	2.29	2.24	2.28	2.24	2.29	2.25	2.28	2.24	2.29	2.24	2.28
Fe1–S2	2.24	2.22	2.23	2.22	2.24	2.23	2.22	2.23	2.21	2.21	2.24	2.20	2.23	2.27	2.30	2.27	2.30
Fe1–S3	2.33	2.38	2.33	2.36	2.32	2.29	2.31	2.29	2.30	2.35	2.35	2.35	2.35	2.27	2.25	2.27	2.25
Fe1–S4	2.31	2.33	2.31	2.32	2.30	2.28	2.31	2.28	2.30	2.32	2.34	2.32	2.34	2.27	2.30	2.28	2.30
Fe1–Fe4	2.71	2.79	2.60	2.77	2.62	2.56	2.58	2.56	2.58	2.75	2.76	2.74	2.76	2.58	2.61	2.54	2.57
Fe1–Fe2	2.73	2.94	2.63	2.93	2.60	2.63	2.64	2.62	2.64	2.92	2.82	2.94	2.82	2.55	2.59	2.57	2.61
Fe1–Fe3	2.70	2.75	2.63	2.74	2.65	2.59	2.61	2.57	2.60	2.74	2.76	2.74	2.77	2.64	2.64	2.63	2.63
Fe3–SG(Cys-63)	2.27	2.22	2.28	2.24	2.30	2.22	2.27	2.21	2.25	2.22	2.25	2.22	2.25	2.21	2.25	2.21	2.25
Fe3–S2	2.32	2.36	2.35	2.36	2.35	2.30	2.34	2.29	2.35	2.36	2.37	2.36	2.38	2.28	2.30	2.30	2.33
Fe3–S4	2.25	2.24	2.23	2.24	2.23	2.21	2.23	2.21	2.23	2.24	2.25	2.25	2.26	2.28	2.31	2.27	2.31
Fe3–S1	2.31	2.35	2.34	2.35	2.35	2.27	2.32	2.27	2.32	2.34	2.35	2.34	2.35	2.25	2.22	2.26	2.22
Fe3–Fe2	2.70	2.77	2.63	2.78	2.66	2.59	2.61	2.59	2.61	2.76	2.76	2.76	2.75	2.57	2.60	2.58	2.62
Fe3–Fe4	2.75	2.81	2.63	2.81	2.66	2.55	2.63	2.53	2.63	2.84	2.81	2.84	2.81	2.63	2.64	2.60	2.63
Fe2–SG(Cys-46)	2.30	2.21	2.26	2.22	2.27	2.21	2.25	2.22	2.25	2.21	2.24	2.21	2.25	2.22	2.27	2.22	2.27
Fe2–S3	2.30	2.38	2.36	2.38	2.37	2.31	2.34	2.32	2.35	2.37	2.38	2.38	2.39	2.25	2.32	2.26	2.32
Fe2–S4	2.29	2.36	2.33	2.35	2.33	2.30	2.33	2.29	2.32	2.36	2.35	2.36	2.35	2.21	2.23	2.21	2.23
Fe2–S1	2.23	2.21	2.23	2.21	2.24	2.23	2.21	2.22	2.19	2.20	2.23	2.19	2.21	2.24	2.30	2.24	2.30
Fe2–Fe4	2.71	2.75	2.59	2.77	2.64	2.58	2.59	2.60	2.60	2.76	2.77	2.77	2.77	2.53	2.68	2.50	2.69
Fe4–SG(Cys-77)	2.27	2.22	2.26	2.23	2.27	2.24	2.28	2.23	2.28	2.25	2.28	2.25	2.28	2.24	2.30	2.22	2.28
Fe4–S2,	2.29	2.34	2.31	2.34	2.31	2.25	2.30	2.25	2.30	2.33	2.34	2.33	2.34	2.20	2.22	2.20	2.22
Fe4–S3	2.28	2.29	2.28	2.27	2.26	2.24	2.28	2.24	2.27	2.30	2.32	2.30	2.32	2.29	2.34	2.27	2.33
Fe4–S1	2.29	2.31	2.29	2.31	2.29	2.24	2.28	2.24	2.28	2.32	2.32	2.32	2.31	2.25	2.31	2.24	2.30
	**MAD**	0.05	0.04	0.05	0.036	0.06	0.04	0.06	0.05	0.05	0.04	0.05	0.04	0.07	0.058	0.07	0.06

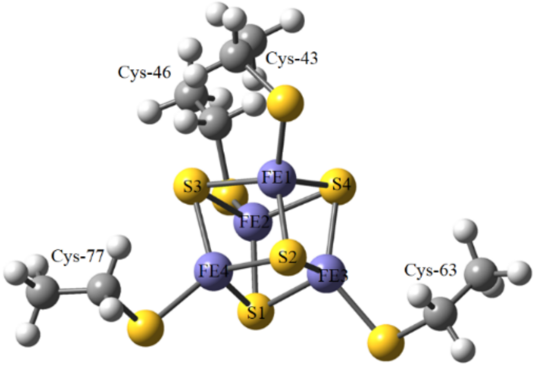

Figure S11. Min QM system for Hip1.

Table S17. Fe–Fe and Fe–S distances of the Min, Int, and Big systems in the optimized structures for Hip2. Atom names are defined in **Figure S12**.

QM system	2HIP, 2.5 Å	Min				Int								Big			
Force field		FF14SB				FF14SB				FF15IPQ				FF14SB			
Surroundings		Fix		Relax		Fix		Relax		Fix		Relax		Fix		Relax	
Oxidation state		Ox	Red	Ox	Red	Ox	Red	Ox	Red	Ox	Red	Ox	Red	Ox	Red	Ox	Red
Fe1–SG(Cys-31)	2.18	2.22	2.27	2.21	2.26	2.28	2.32	2.25	2.28	2.26	2.30	2.25	2.29	2.22	2.26	2.23	2.27
Fe1–S2	2.22	2.22	2.26	2.22	2.27	2.24	2.22	2.22	2.20	2.21	2.24	2.19	2.23	2.21	2.24	2.20	2.23
Fe1–S3	2.22	2.38	2.36	2.37	2.36	2.30	2.32	2.30	2.32	2.36	2.36	2.35	2.36	2.37	2.38	2.37	2.36
Fe1–S4	2.22	2.34	2.35	2.32	2.34	2.30	2.32	2.28	2.31	2.33	2.34	2.31	2.34	2.33	2.34	2.33	2.33
Fe1–Fe2	2.64	2.96	2.79	2.95	2.78	2.63	2.63	2.64	2.63	2.93	2.81	2.94	2.81	2.90	2.82	2.98	2.81
Fe1–Fe3	2.66	2.76	2.77	2.74	2.78	2.62	2.64	2.58	2.61	2.76	2.78	2.78	2.79	2.75	2.78	2.77	2.78
Fe1–Fe4	2.65	2.78	2.77	2.75	2.77	2.55	2.57	2.55	2.56	2.74	2.75	2.69	2.74	2.73	2.75	2.75	2.76
Fe2–SG(Cys-34)	2.42	2.23	2.28	2.24	2.29	2.24	2.28	2.25	2.29	2.22	2.25	2.23	2.25	2.25	2.29	2.24	2.29
Fe2–S1	2.22	2.21	2.25	2.23	2.27	2.22	2.20	2.24	2.21	2.20	2.23	2.21	2.23	2.21	2.23	2.20	2.23
Fe2–S3	2.22	2.39	2.39	2.38	2.39	2.32	2.36	2.35	2.37	2.38	2.39	2.42	2.39	2.34	2.36	2.36	2.37
Fe2–S4	2.22	2.37	2.36	2.35	2.35	2.31	2.34	2.31	2.33	2.36	2.35	2.35	2.35	2.35	2.35	2.35	2.34
Fe2–Fe3	2.65	2.77	2.76	2.78	2.76	2.61	2.62	2.62	2.64	2.74	2.74	2.71	2.74	2.74	2.75	2.76	2.76
Fe2–Fe4	2.66	2.76	2.77	2.77	2.77	2.59	2.60	2.61	2.60	2.77	2.78	2.82	2.79	2.77	2.78	2.77	2.78
Fe3–SG(Cys-48)	2.09	2.23	2.28	2.25	2.31	2.23	2.25	2.21	2.24	2.23	2.27	2.24	2.28	2.22	2.25	2.23	2.25
Fe3–S1	2.22	2.34	2.36	2.35	2.36	2.27	2.32	2.28	2.33	2.34	2.34	2.30	2.34	2.33	2.34	2.34	2.35
Fe3–S2	2.22	2.36	2.38	2.35	2.37	2.31	2.36	2.30	2.35	2.37	2.38	2.39	2.38	2.36	2.37	2.36	2.37
Fe3–S4	2.22	2.26	2.26	2.25	2.25	2.23	2.25	2.22	2.24	2.26	2.26	2.27	2.27	2.26	2.27	2.25	2.26
Fe3–Fe4	2.67	2.80	2.78	2.82	2.79	2.56	2.65	2.54	2.61	2.84	2.81	2.84	2.80	2.86	2.83	2.82	2.80
Fe4–SG(Cys-64)	2.02	2.21	2.25	2.23	2.28	2.24	2.28	2.25	2.28	2.25	2.28	2.26	2.29	2.25	2.28	2.25	2.27
Fe4–S1	2.22	2.31	2.32	2.31	2.32	2.25	2.29	2.24	2.28	2.33	2.33	2.30	2.31	2.33	2.33	2.34	2.33
Fe4–S2	2.22	2.35	2.37	2.34	2.35	2.26	2.30	2.25	2.29	2.33	2.34	2.32	2.34	2.33	2.34	2.34	2.34
Fe4–S3	2.22	2.28	2.30	2.26	2.29	2.24	2.28	2.26	2.30	2.28	2.31	2.28	2.32	2.28	2.30	2.29	2.31
	**MAD**	0.12	0.12	0.12	0.12	0.07	0.09	0.07	0.08	0.12	0.12	0.12	0.12	0.11	0.12	0.12	0.12

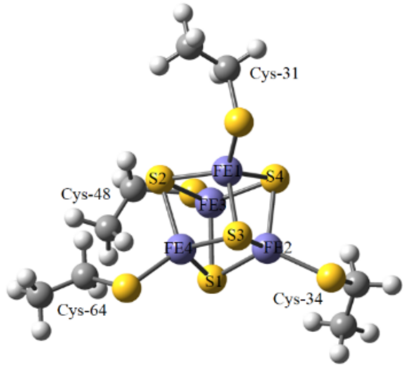

Figure S12. Min QM system for Hip2.

Table S18. Mulliken spin populations of the various QM/MM structures. The first 16 results were obtained for optimized structures, whereas the remaining six were obtained from single-point calculations on structures optimized with TPSS/def2-SV(P).

Method		QM/MM; Opt																QM/MM; sp					
Theory level		TPSS/def2-SV(P)																TPSS/def2-TZVPD		B3LYP/def2-TZVPD		B3LYP/def2-SV(P)	
QM system		Min				Int								Big				Min		Min		Min	
Force field		FF14SB				FF14SB				FF15IPQ				FF14SB				FF14SB		FF14SB		FF14SB	
Surroundings		Fix		Relax		Fix		Relax		Fix		Relax		Fix		Relax		Fix		Fix		Fix	
Oxidation state		Ox	Red	Ox	Red	Ox	Red	Ox	Red	Ox	Red	Ox	Red	Ox	Red	Ox	Red	Ox	Red	Ox	Red	Ox	Red
Rub1	Fe	3.9	3.6	3.9	3.7	3.9	3.6	3.9	3.6	3.9	3.6	3.9	3.6	3.9	3.6	3.9	3.6	3.8	3.5	3.9	3.6	4.0	3.7
Rub2	Fe	3.9	3.6	3.9	3.6	3.9	3.6	3.9	3.6	3.9	3.6	3.9	3.6	3.9	3.6	3.9	3.6	3.8	3.4	3.9	3.5	4.0	3.7
2Fd1	Fe1	-3.6	-3.3	-3.7	-3.3	-3.6	-3.3	-3.6	-3.3	-3.6	-3.3	-3.6	-3.3	-3.6	-3.3	-3.6	-3.3	-3.6	-3.5	-3.8	-3.5	-3.9	-3.6
	Fe2	3.6	3.7	3.6	3.7	3.6	3.7	3.6	3.7	3.6	3.7	3.6	3.6	3.6	3.6	3.6	3.6	3.6	3.6	3.8	3.8	3.8	3.9
2Fd2	Fe1	-3.7	-3.4	-3.7	-3.4	-3.6	-3.3	-3.6	-3.3	-3.6	-3.3	-3.6	-3.2	-3.5	-3.3	-3.5	-3.2	3.6	-3.3	3.8	-3.5	-3.9	-3.6
	Fe2	3.7	3.8	3.7	3.7	3.6	3.7	3.6	3.7	3.6	3.7	3.6	3.7	3.6	3.7	3.6	3.7	-3.6	3.6	-3.8	3.8	3.9	3.9
Rieske	Fe1	3.5	3.6	3.6	3.7	3.5	3.6	3.5	3.6	3.5	3.6	3.5	3.6	-3.6	-3.6	-3.5	-3.6	3.6	-3.0	3.8	-3.4	3.8	3.8
	Fe2	-3.6	-3.3	-3.7	-3.3	-3.7	-3.2	-3.7	-3.3	-3.6	-3.2	-3.7	-3.3	3.6	3.3	3.6	3.2	-3.8	3.6	-4.0	3.8	-3.9	-3.5
3Fd1	Fe1	2.5	-3.5	-3.4	-3.5	2.5	-3.4	2.3	-3.4	2.5	-3.4	2.2	-3.4	2.4	-3.4	2.0	-3.4	1.3	-3.6	1.3	-3.8	2.9	-3.8
	Fe3	2.0	3.5	2.8	3.5	1.9	3.4	2.2	3.4	2.0	3.4	2.3	3.4	2.0	3.4	2.4	3.5	3.4	3.5	3.8	3.7	2.7	3.7
	Fe4	-3.5	3.6	1.8	3.6	-3.4	3.5	-3.4	3.5	-3.4	3.5	-3.4	3.5	-3.4	3.5	-3.4	3.5	-3.5	3.5	-3.8	3.6	-3.8	3.7
3Fd2	Fe1	-3.4	3.5	2.7	3.5	-3.3	3.5	-3.3	3.5	-3.3	3.5	-3.3	3.5	2.6	3.5	2.5	3.5	3.5	3.5	3.8	3.7	-3.8	3.7
	Fe3	2.6	-3.5	1.7	-3.5	2.8	-3.4	2.9	-3.4	2.8	-3.4	2.9	-3.4	-3.3	-3.4	-3.4	-3.4	1.1	-3.6	1.1	-3.8	2.9	-3.8
	Fe4	2.0	3.6	-3.4	3.6	1.6	3.5	1.5	3.5	1.6	3.5	1.5	3.5	1.7	3.5	1.9	3.5	-3.5	3.5	-3.8	3.7	2.7	3.7
4Fd1	Fe1	3.4	-3.4	3.4	-3.5	3.3	-3.4	3.3	-3.4	3.4	-3.4	3.4	-3.3	-3.3	-3.4	-3.2	-3.3	-3.4	-3.4	3.6	-3.6	3.7	-3.7
	Fe2	-3.3	3.0	-3.4	3.0	-3.3	2.9	-3.3	2.9	-3.4	3.0	-3.4	3.1	3.3	3.0	3.3	2.9	3.4	3.1	-3.6	3.4	-3.6	3.4
	Fe3	-3.4	3.3	-3.4	3.3	-3.3	3.2	-3.3	3.2	-3.5	3.2	-3.4	3.2	3.3	3.1	3.2	3.2	3.4	3.2	-3.7	3.5	-3.7	3.6
	Fe4	3.4	-3.5	3.4	-3.5	3.3	-3.4	3.3	-3.4	3.4	-3.4	3.4	-3.4	-3.3	-3.4	-3.3	-3.4	-3.4	-3.4	3.7	-3.7	3.7	-3.7
4Fd2	Fe1	3.5	-3.6	3.5	-3.5	3.3	-3.4	3.3	-3.4	-3.5	-3.5	-3.5	-3.5	3.4	-3.4	3.4	-3.4	3.4	-3.4	3.6	-3.6	3.7	-3.7
	Fe2	3.5	-3.6	3.5	-3.5	3.3	-3.4	3.3	-3.4	-3.4	-3.5	-3.4	-3.5	3.4	-3.5	3.5	-3.5	3.4	-3.5	3.7	-3.7	3.7	-3.8
	Fe3	-3.4	3.1	-3.4	3.1	-3.3	3.0	-3.3	2.9	3.4	3.2	3.4	3.3	-3.4	3.2	-3.5	3.3	-3.4	3.1	-3.6	3.5	-3.7	3.5
	Fe4	-3.5	3.4	-3.5	3.4	-3.3	3.2	-3.3	3.3	3.5	3.4	3.5	3.4	-3.4	3.3	-3.5	3.2	-3.5	3.2	-3.7	3.5	-3.7	3.6
4Fd3	Fe1	-3.5	3.3	-3.5	3.2	-3.4	2.9	-3.4	2.9	-3.4	3.2	-3.4	3.2	-3.4	3.1	-3.4	3.0	-3.4	3.1	-3.6	3.5	-3.7	3.6
	Fe2	3.4	-3.5	3.4	-3.5	3.4	-3.4	3.4	-3.4	3.4	-3.4	3.4	-3.4	3.4	-3.5	3.4	-3.4	3.3	-3.4	3.6	-3.6	3.7	-3.7
	Fe3	-3.5	3.3	-3.5	3.3	-3.4	3.2	-3.4	3.2	-3.4	3.1	-3.4	3.2	-3.4	3.3	-3.4	3.3	-3.4	3.1	-3.7	3.5	-3.7	3.6
	Fe4	3.5	-3.6	3.5	-3.6	3.5	-3.4	3.5	-3.4	3.5	-3.4	3.5	-3.4	3.5	-3.5	3.4	-3.5	3.5	-3.5	3.7	-3.7	3.8	-3.8
Hip1	Fe1	3.3	3.3	3.3	3.3	2.8	3.3	2.8	3.3	3.3	3.5	3.3	3.4	3.1	3.3	3.1	3.3	3.4	3.5	3.7	3.7	3.7	3.6
	Fe2	-3.5	-3.3	-3.6	-3.3	-3.1	-3.2	-3.1	-3.2	-3.5	-3.4	-3.5	-3.4	-2.6	-3.2	-2.7	-3.2	-3.6	-3.4	-3.8	-3.6	-3.8	-3.6
	Fe3	-3.6	-3.3	-3.6	-3.3	-3.1	-3.3	-3.1	-3.2	-3.5	-3.4	-3.5	-3.4	-2.8	-3.3	-2.7	-3.3	-3.6	-3.5	-3.8	-3.7	-3.8	-3.6
	Fe4	3.3	3.3	3.3	3.4	2.8	3.3	2.8	3.3	3.3	3.4	3.3	3.4	3.1	3.2	3.1	3.2	3.3	3.4	3.6	3.6	3.6	3.6
Hip2	Fe1	-3.6	3.5	-3.6	3.5	-3.2	3.3	-3.1	3.2	-3.5	3.4	-3.5	3.4	-3.5	3.4	-3.5	3.4	-3.6	3.5	-3.9	3.7	-3.8	3.7
	Fe2	-3.6	3.5	-3.6	3.5	-3.2	3.3	-3.2	3.3	-3.5	3.4	-3.5	3.4	-3.5	3.4	-3.5	3.4	-3.6	3.4	-3.8	3.6	-3.8	3.7
	Fe3	3.3	-3.5	3.3	-3.5	2.9	-3.3	2.9	-3.3	3.3	-3.4	3.3	-3.4	3.3	-3.4	3.3	-3.4	3.4	-3.5	3.7	-3.7	3.6	-3.7
	Fe4	3.3	-3.5	3.3	-3.5	2.9	-3.3	2.9	-3.3	3.4	-3.5	3.4	-3.5	3.3	-3.4	3.3	-3.4	3.4	-3.5	3.7	-3.7	3.7	-3.7

Table S19. Approximate calculation time in minutes for the QM/MM calculations (on Intel Xeon E5-2650 v3 processors with 20 cores).

Theory level	TPSS/def2-SV(P)								TPSS/def2-TZVPD						B3LYP/ TZVPD		B3LYP/def2-SV(P)							
QM System	Min		Int				Big		Min		Int				Min		Min		Int				Big	
Force field	FF14SB		FF14SB		FF15IPQ		FF14SB		FF14SB		FF14SB		FF15IPQ		FF14SB		FF14SB		FF14SB		FF15IPQ		FF14SB	
System 2	Fix	Relax	Fix	Relax	Fix	Relax	Fix	Relax	Fix	Relax	Fix	Relax	Fix	Relax	Fix	Relax	Fix	Relax	Fix	Relax	Fix	Relax	Fix	Relax
Rub1	2.0	3.5	73.5	82.5	74.6	83.6	223.9	289.5	0.2	0.2	16.0	16.2	10.0	18.0	3.6	3.4	0.1	0.1	1.3	1.3	1.6	1.6	4.4	4.4
Rub2	2.5	4.0	74.7	83.7	92.9	101.1	164.7	217.5	0.2	0.2	10.0	20.8	12.0	23.0	3.4	3.5	0.1	0.1	1.5	1.4	1.8	1.8	4.8	4.8
2Fd1	6.0	9.0	102.6	120.6	107.6	125.6	434.7	577.5	2.5	3.5	73.7	81.9	60.0	60.0	3.6	4.6	0.2	0.1	4.1	3.5	3.4	4.5	11.1	10.8
2Fd2	9.0	12.0	142.7	160.7	353.4	371.4	474.9	600.0	1.2	3.0	117.6	34.0	60.0	54.0	3.5	57.8	0.1	0.1	3.4	3.3	3.1	3.0	11.3	29.9
Rieske	7.5	9.5	300.0	330.0	259.9	277.9	265.9	432.0	2.9	4.0	150.0	106.0	63.0	30.0	3.9	5.1	0.4	0.4	4.8	4.1	5.9	5.6	29.8	18.2
3Fd1	15.0	19.5	213.0	249.0	156.3	192.3	296.5	504.0	2.0	3.8	95.1	56.2	60.0	33.0	12.5	49.5	0.4	1.0	8.5	7.1	9.6	7.4	18.5	17.7
3Fd2	13.0	18.0	148.3	184.3	161.4	197.4	360.7	580.0	3.1	2.4	63.0	8.2	43.2	55.0	24.2	13.1	0.4	0.4	5.1	9.1	6.6	10.0	43.2	29.4
4Fd1	18.0	22.5	169.1	214.1	180.0	225.0	624.0	648.0	1.3	12.0	237.3	151.8	50.0	240.0	72.3	83.0	0.4	0.5	6.0	5.4	6.3	6.5	19.3	20.2
4Fd2	19.5	24.0	191.9	236.9	128.7	173.7	586.0	600.0	3.2	5.4	89.1	47.9	49.8	108.0	78.0	87.0	0.4	0.4	4.6	4.7	5.1	6.3	20.4	18.6
4Fd3	22.0	24.5	337.5	382.5	312.2	357.2	1079.0	1260.0	1.4	5.6	62.0	83.3	180.0	127.0	87.5	93.0	0.3	0.4	6.0	5.4	5.4	5.1	23.0	22.9
Hip1	18.5	23.0	140.5	185.5	135.0	180.0	650.0	1008.0	7.5	9.0	90.2	167.0	83.0	34.3	93.0	90.0	0.4	0.4	6.2	5.1	5.3	4.7	37.3	38.1
Hip2	22.0	24.5	111.9	156.9	118.0	163.0	389.6	648.0	4.0	5.0	34.7	102.3	61.3	42.0	17.5	36.0	0.4	0.4	6.2	4.8	4.6	5.3	32.9	34.1

Table S20. Approximate calculation time in minutes for QM+COSMO calculations with fixed surroundings, TPSS/def2-SV(P) level (on Intel Xeon E5-2650 v3 processors with 20 cores; some calculations are performed on a single core, and those values were adjusted for 20 cores).

QM system	Min			Int						Big		
Force field	FF14SB			FF14SB			FF15IPQ			FF14SB		
ε	4	20	80	4	20	80	4	20	80	4	20	80
Rub1	0.04	0.1	0.1	0.6	1.1	1.1	2.0	2.0	2.0	3.1	4.9	4.9
Rub2	0.05	0.1	0.1	0.7	1.2	1.1	0.9	1.4	1.4	0.6	1.1	1.1
2Fd1	0.05	0.1	0.1	2.0	2.8	2.9	3.0	4.0	4.0	3.9	6.2	6.1
2Fd2	0.05	0.1	0.1	1.5	2.1	2.1	3.0	4.0	4.0	2.8	3.9	4.4
Rieske	0.15	0.2	0.2	4.0	7.0	7.0	5.0	8.0	8.0	14.6	18.6	18.3
3Fd1	0.35	0.4	0.4	0.4	1.7	1.8	5.0	7.0	8.0	8.5	11.6	12.2
3Fd2	0.10	0.1	0.2	1.7	2.5	2.7	3.0	6.0	6.0	0.9	1.9	1.8
4Fd1	0.10	0.2	0.2	1.9	3.2	3.4	5.0	7.0	8.0	7.3	11.2	12.6
4Fd2	0.10	0.2	0.2	1.4	2.8	2.8	4.0	6.0	6.0	5.5	9.4	10.5
4Fd3	0.10	0.2	0.2	1.5	2.6	2.8	3.0	6.0	6.0	26.3	34.3	39.4
Hip1	0.10	0.2	0.2	3.2	4.2	4.2	7.0	9.0	9.0	17.5	24.4	23.3
Hip2	0.10	0.2	0.2	3.2	4.3	3.9	6.0	9.0	9.0	5.2	7.0	7.0

Table S21. Approximate calculation time in minutes for the QTCP calculations, TPSS/def2-SV(P) level (on Intel Xeon E5-2650 v3 processors with 20 cores. LR is the long-range corrections, Born, Generalised Born (GB) or Ewald. Note that the latter two are corrections, run after the Born calculations, and are based on the same MD simulations.

QM system	Min			Int					
Force field	FF14SB			FF14SB			FF15IPQ		
LR	Born	GB	Ewald	Born	GB	Ewald	Born	GB	Ewald
Rub1	51.0	21.0	0.5	90.0	24.0	0.5	106.6	24.0	0.5
Rub2	51.0	21.0	0.5	100.0	24.5	0.6	116.8	24.7	0.6
2Fd1	54.9	24.0	0.6	160.0	25.0	0.6	172.5	25.2	0.5
2Fd2	66.0	45.0	0.8	360.0	48.0	0.8	375.0	51.0	1.0
Rieske	54.0	42.0	0.7	220.0	48.0	0.9	229.3	48.1	0.7
3Fd1	57.5	49.5	0.7	288.0	48.0	0.8	303.0	50.0	0.7
3Fd2	79.0	50.0	0.7	429.0	51.0	0.8	438.0	51.0	0.7
4Fd1	90.3	64.2	0.8	305.0	55.5	0.9	318.7	61.5	0.8
4Fd2	78.5	51.0	0.7	358.0	46.0	0.8	367.1	49.3	0.8
4Fd3	83.5	51.0	0.7	360.0	48.6	0.8	368.1	49.7	0.8
Hip1	90.0	63.0	0.8	325.0	61.5	0.9	335.0	61.3	0.8
Hip2	77.0	51.0	0.7	305.0	51.5	0.7	315.0	52.2	0.7
